# Supplementary material for: Genome-Wide Identification and Expression Analysis Reveals the B3 Superfamily Involved in Embryogenesis and Hormone Responses in Dimocarpus longan Lour
Source: Int J Mol Sci. 2023 Dec 21;25(1):127. doi: 10.3390/ijms25010127 (PMC10779397; doi:10.3390/ijms25010127)
Supplement: Supplementary file 1 [file ijms-25-00127-s001.zip › supplementary materials.pdf]

Supplementary Table S1 Primers Used in Vector Constructions and qRT-PCR

Primer Name	Primer sequence（5' to 3'）	Function
pRI101-LEC2-F	GGGGTACCATGGAAAACACCTTCTCAAC	Construction of *DlB3*:EGFP
pRI101-LEC2-R	GGAATTCTAGAGAAAAATTATACTCATTAACAT	
pRI101-FUS3-F	GCTCTAGAATGATGGACCAAGCGGCT	
pRI101-FUS3-R	CGGAATTCTTAGAAGTCATCAAGAGA	
DlARF5-qF	TGCTGGTCCTCTTGTTTCCT	qRT-PCR
DlARF5-qR	AATTTGGGTCGTTGCTGTCC	
DlARF16-qF	AAAAGGGAGGGATTGGTGGT	
DlARF16-qR	CGCTGACATTTGCCCTGAAA	
DlTEM1-qF	ATCGCATTCCCAGTGTTGTG	
DlTEM1-qR	TTTGCGCCACTTTGAACAGT	
DlVAL2-qF	TATGACATCGCTGCACAACG	
DlVAL2-qR	TCAACGATCTCAGCCTTGGA	
DlLEC2-qF	ACCCACCAACCACAGTAGCA	
DlLEC2-qR	TGATGGAGTGTTTGAGCCG	
DlFUS3-qF	GGAGACTTTGTTAGCACCCA	
DlFUS3-qR	CTGTCCGAAGCCAATGTT	
DlREM9-qF	AAATCCGTTCTTTACTTCCTGC	
DlREM9-qR	TCCAACCCGAATCAAACC	
DlREM40-qF	TATCTGTGGCGGTTCTGTGT	
DlREM40-qR	AACAGCCCACTCTCTTCCAT	
DlUBQ-qF	GCCGACTACAACATCCAGAAG	qRT-PCR (Reference gene)
DlUBQ-qR	GCTTGGTGTAGGTCTTCTTCTT	
DlEF1α-qF	GATAATTCCCACCAAGCCCAT	
DlEF1α-qR	GGGTCCTTCTTCTCAACACTCT	
DlACTB-qF	TGCTATCCTTCGGTTGGACC	
DlACTB-qR	CGGACGATTTCCCGTTCAG	

Supplementary Table S2 Analysis of transcription start site of *DlB3s*

Gene name	Score	Position	Core promoter sequence	Transcription initiation site
DlARF1	0.91	1260-1310	ATGTAATTTTTCTATATGTGGGAGCACT ACCAACGTACCAAGTTTTTTGA	A
DlARF2	0.87	271-321	CACCTCACACTATAAATAGGGATAGAAA ATCATTGTAAACGGACATAAGC	G
	0.82	663-713	ATGGTTAGCATATATAAACAATACTAAT GACATTGATAGTAAGGTACAAA	A
	1	1355-1405	AAGTCATATATATATATACACACCAAGA CACACTAAGTTTAGTTATTATT	A
	0.86	1435-1485	GAAATATTCATATATACACACCTCCATT	T

			ATTAGAAGAAAATAATTAACTA	
	0.96	1834-1884	AAACTCTACATAAAAAACACCACTCATC GTCCTTTCGCTTCTTCATCCAT	C
	0.98	686-736	CTTTAATATATATAAATAATCGGCTTTA AATGTTCCCTTCTTTATATGAA	T
	0.92	1008-1058	CTAATCCTTATAAAAAAATGTTCCCTTA ACTTAATTGGTCTTAAAGGCTT	T
DlARF3	0.95	1428-1478	AGTGAAGCACTAAAAAGCTCGCAGTGGT GTCTCCTTCTCTCTTTCTCTGT	C
	0.89	1576-1626	AACATATATATATATATGTATATATACA AAAAAGAAAAACAAAGAGCGAT	A
	0.91	1780-1830	AAGCAAAAAATAAAAGAGGGAGAGAGA AAAAGGGGTCTTTACTCCTTTTC	A
	0.92	1884-1934	TTTTTGCCTCTAAAAATGCAGTCATGAG CGTCTCTTTCTTGTCCACTCTT	G
	0.9	36-86	TCGCCGCCTTTATCAGACGGCCACAATT CGCTTTCGACTGAGTGACGTAC	A
	0.89	295-345	GACAGACAAATAAAAATGCGTTCGTCAT TTCTGTTTTATAAAATTATATT	A
DlARF4	0.95	483-533	GTGATCCATATATAAATGAACACCGATA TTATATATTATAAAAGGAACAA	A
	0.87	532-582	AAACATGTTATATAAAAAATTCATTGAA TTATTGTGTTTATCCAACCTTT	T
	0.95	647-697	ATGATTCATATATAAATAAACACTGATA GTGTAAATTATAAAAGAAATAA	A
DlARF5	0.86	998-1048	AATCCACGTGCTAAAATGCGCCATCTCT CTCTCTTTTTATTTTGGTTAAT	T
	0.86	1071-1121	TTTTTCTCTGTATATAACGTACTGCTGTT GTTGGTGTTCTGTTTTCGTGT	G
DlARF6	0.93	1553-1603	TGTTTATGTTTATAAACTTGGAGTTGGTC AAGCAAGAGTCCAATTGAGAT	C
	0.96	939-989	AATAAAATATATATACACATGCACATGT GCATGAGTGAGCATGCGTGCAC	A
DlARF7	0.96	1211-1261	GGATGTCTATATAAAATAAAAACGACAA ATTAGAGAAACCAGCCAAAGAA	A
	1	1497-1547	CAAACAAAAAAAAAAAAGGGCGGAGAA GACCAGAGAGCGATGCATGGCAT	T
DlARF8	0.99	666-716	TATTTTAGTTTTTAAAAGGGCTAAAAAA CTGTTTTAAGGACTAAAAAACC	C
	0.98	1450-1500	GTCAAATCAATAAAAAAGAACGGACGG TCAGCGTAGTCTCGTACGGATAC	G
DlARF9	0.91	44-94	CAAATAAACATATACAGGTGGGGCCCTA ACCCGACAATAACTGAACCACG	C
	0.9	266-316	TTAAATTAAATAAAATACGACCCTAAAA	A

			TTCTGCCGGGTTAACTTGAGCC	
	0.89	276-326	TAAAATACGACCCTAAAATTCTGCCGGG<br>TTAACTTGAGCCAACCCTGTTG	A
	0.85	376-426	AACGTCCCAATTTAAAGCATCGGCGCAT<br>CCGTTACTCCTCACCACACGCA	A
	0.92	674-724	TTCTAATTTATATAAAAAAATGTACAGA<br>CTTGTTTTTCTTGTAATATGAG	G
	0.98	1298-1348	TATTCCTATAAATAAATGCACAAAACGG<br>CCGTCATGAACCGTTACGAAAT	G
	0.94	1483-1533	CAACAGTGTGGAAAAAAAAGCAGACAA<br>AAGCAACAAAGCCACCCCCACCA	A
	0.99	1540-1590	AGTCGCGTAATATAAGACGCGCGCGAAC<br>CGGTCTCTCTTCTTGTTGGATG	T
	0.99	1898-1948	ATTTTGTGTATATAAATAAGTAGAGATT<br>GAAAATTAGGGTATAGAGATAC	A
	0.83	353-403	AACATTATTATATAAAAGCAAAATTTTA<br>TCTCCTCTTATCTTAAACTTTT	T
	0.83	710-760	CAGGTGCTTATAAAACTATGGTTTACAA<br>GGGAAACCAGATATTCCTTGTT	A
DlARF10	0.92	1508-1558	AACGTCGCCAATTAAATTCCCCGTCTGC<br>CTCTTAACCAGTGGACACGTAC	G
	1	1598-1648	TATTTATCTATATATATAGAGCGAAAAA<br>AAGCAGTGATCGTACGAACTGT	T
	0.81	1613-1663	ATAGAGCGAAAAAAAGCAGTGATCGTA<br>CGAACTGTAGGTTAATGGCAGCA	A
DlARF11	0.95	268-318	TAATGAAGGAAAAAAAAAAGCCGTTTA<br>TAACCTTTTAATGAAGTTTTTTT	A
	0.81	1845-1895	GAAGGTATGGTATATATTTGGTGTAGAG<br>AGCAAGAAGTGATACAAGGCTT	T
	0.96	791-841	TTGTAACCTGTATATGATTAGCCTCGCG<br>CACATAGCGCTGATGTGATCTA	A
DlARF12	0.97	1130-1180	TTAAGGCTTGTAAAAAATTACCTCGGTG<br>TGCTAATGACCCACTTGGAATA	A
	0.85	1238-1288	TTTGTTCAGGATTAAACCCGCCACTTGG<br>GGCAGGGGTAGGTTCTGATTGA	T
	0.86	575-625	CTTTTTTATATATAAAAACTTAAAAAAA<br>AAAATAATAAAATAGAAAACCA	T
DlARF13	0.99	1460-1510	TTGTTTATATGTGTATATAATCACGATCT<br>AGTGCTCGATCAAATGTGTTT	A
	0.82	1601-1651	GAAAATCTGTTAAAAAATTGCTGTTTTT<br>GCTCCAAAAAAAGGGGTCCATG	G
	1	1623-1673	GTTTTTGCTCCAAAAAAAGGGGTCCATG<br>GATTTGTTGCACGGCTTTGGGC	G
DlARF14	0.86	424-474	TTAATAGCACTATATTGGACGCGCGTGG	G

			AAGCTCAAAAGTGTACACGACG	
	0.97	501-551	TGCAGAGTTTAAAAAACGACACCCTCAC<br>TAAAATTAAATCACCAACCACG	A
	0.87	546-596	CCACGCGCTTTAAAGAGGCAGTACCAGA<br>GATAGAGTTTTAAGAGTTACAG	A
	1	972-1022	GCACACAGTAAAAAAAAGAGCGGGGGG<br>GTGGGTTGAACTTTAAAGTGGGG	T
	0.84	1000-1050	TGGGTTGAACTTTAAAGTGGGGTCGTTT<br>TGTTGTGTTGTGTCCGTGTGTG	T
	0.9	1383-1433	GAGAGAGGGATATAAAAATCTGTGAAT<br>CAGATAGATGATTTGGTGGTCAT	T
	0.92	397-447	GCGTAGACTCTTTATTGGGCCCACAAAC<br>ACTCCTCCATCCATGTAGTGCG	A
DlARF15	0.81	664-714	CGAGTTCATACAAAAAATCTCGCGAGAC<br>ATTTAAATTTACAAAAATATCG	A
	0.94	934-984	ACACAAAAGGAAAAAGAGGGGGCTGCT<br>AATACTACAGTGCAACACTAAAA	A
	0.98	1432-1482	TTAAGTTACGTAAAAATATCGCGCGTAA<br>CAGGAGACGCGGGAGAGGAGAA	G
	0.85	1479-1529	GAATTATGTCAACAAAAGGGGTGCGCGT<br>GGTAGTGATCTGAAAAAGAAAG	A
DlARF16	1	1627-1677	AGCATCGTAAAATAAAAGGCGCACACA<br>CAATTCATTTCTCATTTCACTAG	A
	0.92	1681-1731	TCTGTTTTTTCAAAAAGCGCTTTTTAACA<br>CTGGACAACGCAACACTCTGT	A
	0.97	512-562	TGTATATGTATATATATATCACATAATTT<br>TAGTCATATATATCCAACAAA	A
	0.97	670-720	AGAAAACAGTTTAAAAAACATGGACTGT<br>CGGCAGTAGGGCATACCAGTAG	A
DlARF17	0.94	1554-1604	TTATTTATTTATTAAAAAGTGCATAATTG<br>ACGTTGATGCCATAATGTTAA	A
	0.88	1672-1722	TTATTTATTTATTAAAAAGTGTGTAATTG<br>ACGTTGATGCCATAATGTTGC	A
	0.98	1758-1808	AGGGTAAAAGTGTAAAAAAGTGGTCTG<br>GGACATGTAGAGCAGGGAGAAAC	A
DlARF19	0.88	747-797	AATAAAAACATATAAGAGCAGTGAAGA<br>AAAATCTTCTTTCCATATGACGT	C
	0.82	1517-1567	AAAAGGCAAATAAAAAAATGGAAACCA<br>AGAGTACCCATTTTTTGATTTGC	T
DlVAL1	0.97	783-833	TATAATTATATATATATAATACCTATATT<br>CTCGCTTATTAATAATTAGGA	A
	0.86	1402-1452	AATTAAAATTAAAAAAAACCGAAGTCA<br>AAAAAGAAAAAAAAAAAAAAAAG	A
DlVAL2	1	1632-1682	TTCAGATATTAATAAACAGCGCGGACAC	G

			ATCCGCGCAAACGCACGCATGG	
DlVAL2-like	0.86	1739-1789	TGGTTGATAATATAAGTAGGGGGATATA CTTTATGCTTGATCTTTTATTT	T
DlVAL3	0.95	38-88	GAACATGTTATATAAAGTGGTATTATAG GATTGGTCAATCAGGGGGCGCC	A
	0.99	150-200	ATATATATATATATATATTTGCTTCCACA GCTACATATAAAATTCTCATT	A
	0.99	616-666	GAACACATTATATAAAATGCTATTACAG CACTGGTCAACCAGTGGGCACC	A
	0.85	721-771	CACTAATATATATATATATATATATTATA GTTTTACCGTCAATGTTTTTC	A
DlVAL3-like	0.96	368-418	GGCTAATACATATAAATCAGGTACGTAG TATCTATCATATTAGTTGAATT	T
	0.9	836-886	ATATATAATTATATATATATATATACAC GCGCGTGCACACACACATGCAC	A
	1	844-894	TTATATATATATATATACACGCGCGTGC ACACACACATGCACTTTTCTAA	A
	0.95	1030-1080	ACTAACTGCTTTAAAAAGTGGTACAAGG AAACTTTTGATTTTTGTTAAAA	T
DlLEC2	0.99	163-213	CCGTCCTCTATTTATAGAGAGGGGGGAC ATGCTACAGTGAAACAGAATAA	A
	0.95	778-828	CTTGTATGGACAAAATGGGCGAACAGTC GGGCGGTCAATCATGGCGGATA	A
	0.91	1061-1111	GATGAGCGGTCACAAAAGAGGCCCTCC GTCAAGGAGGCGAATTGGAAGAG	A
	0.92	1235-1285	AGAAACCTACTAAAAATAGGGGATGGTT ACTCTTTTTTTTTTAATTTTTT	T
DlFUS3	0.99	184-234	TTTTATATATATATATATATAGTCATTAC TGCCTTAACTTTTTAATTGTT	T
	0.86	368-418	TTATTATATATATAATATAAACCCTAAA TGAGTTTTGCTAAAGTAGATTT	A
	0.89	999-1049	TATTTTTGTTTTAAAAAAAGTCATAAAG TGTCTAACAGGTACGAGCAACA	A
DlABI3	0.91	199-249	ACGGAAATAATATAAAGACACGGAAAT AGCAGCATCCAATAGGGCAGTGA	A
	0.9	675-725	CACAACGTTTTTAAACACCGCTATGGTA TGAAATGACTCTAATGAGGGCT	A
	0.85	899-949	AAAGATTATATATAAATAACTTAAATTC AAACACTAAATGTTGGTATTTC	T
	0.84	1115-1165	TTATGACTCATAAAAGGCTGAGGCACCG TTGGTATGTACCATTGAGAAGA	A
	0.94	1339-1389	TTATTGTGTGTATAAAACCCCAAAGAAC TGATTTGAACGTTAAAGAAGTT	T
	0.97	1459-1509	TGGTTGTCTTCATAAAGGGGTCGTCTCTC	T

			TCAAGTACAATTTGCTTTCCC	
	0.84	1353-1403	TGGTGATATCATATATATTTCCAAGTAT AGGCATGAATCCATTCTTCATT	A
DlRAV1	0.87	1572-1622	TGTTGGTGCAGTATAAGTGATCCCAATT CATCATTCATCAATTTTGAACT	A
	0.94	1777-1827	TGGCTGAGTCTATAACTTCTGGAGGCTG CTCGGTGGTTTCTTGATAATTC	T
	0.8	291-341	TTACAAGTTATAAAAAAACCCTTAATTA AAATTATATACTCTATAAGCAT	C
DlRAV2	0.98	335-385	AAGCATTTTTTTAATAAGGGGTGTAAAA GACATTTTAACTTGTAGAAGGA	T
	0.96	809-859	ATATATATATATATATATTTAGACACAC AAATTTTACTGACTTTTGACTT	C
	0.97	925-975	CTGAGCAGCTTTATAATATAACCCATGC TGAGAGCCACACATTTACAACT	A
	0.86	808-858	CCAATTACATTAAAAAAGGAGATGGTTT TTTTGTTTGAAATAAAAAAGGA	T
DlTEM1	0.88	838-888	TTGTTTGAAATAAAAAAGGACATGGTTT TGGATCCAGAGAATATTTATAG	A
	0.91	1438-1488	TGATAAAAAAAATATAAAAAGAGATAG ACAGAGAGAGGCCATGCTAGTGG	A
	0.88	1870-1920	TAAATACATATATATATTTGTACTCAAA ACGAATCCTCAAACAAATCTCT	A
	1	949-999	TAATATATATATATATATATATGAGAAGGT TTCTCCTTGGTTGGTTTTGTCA	G
DlTEM2	1	1288-1338	ACGATTATTTATTAAAAAGCGGGCCTGC ACGCTTGCACACATCTGCCTTT	A
	0.88	1335-1385	TTTTGTGGCTAAATAAGTTGCATTTTACA TACAGTGCTGCACCACACATA	A
DlNGA1	1	622-672	TGGGTGAACATATAAAAACAGCTGGCGT AGATCAGGACCTTATCACTCAC	T
	0.98	707-757	AAAATATGTGTATAACACCCAGGATGAT GACAATGACAAGCTCACATGAG	C
	0.81	163-213	GTAGGTAATTTATAACTTGGGAAGTGAG GAGAGGAAACTACCAGATATAG	C
	0.93	798-848	GATTTTAATTTAAAATAATGGCAGGTAT AGTAGGAGGTATACGTATATAT	A
DlNGA2	0.99	833-883	GGTATACGTATATATAAATATAGTTACG GAAATGTTCGGTAGTGTTTTGA	A
	0.91	1224-1274	TCCGACTATATATATATTACTAAAATTG AGATAGAGAAACACCCCGAGGC	A
	0.89	1224-1274	AGAAGATATATATATATATATATATATG TCTATAAATATGAAAAAAGCCA	A
	0.98	1807-1857	TATATATATATATATATATGTCTATAAAT	C

			ATGAAAAAAGCCAAGAGTAAG	
	0.98	1824-1874	ATGTCTATAAATATGAAAAAAGCCAAGA<br>GTAAGAAGAGCCGTTGGATGAA	G
DlNGA3	0.96	148-198	AACATGAATCTATATAATTGGGAATGTT<br>TATCCTTAATTACCCATAGTTT	C
	1	1860-1910	ATAAAATTCATTAAAAAGCCCACCTTGT<br>ATTCTTTGTTTTCTCCCTTTTC	C
DlREM1	0.96	1268-1318	TCCCATTCTTTATAAATAGGGGTGAATTT<br>CATTGTAAAATGACGGATTCT	G
	0.8	413-463	CTTCTTTGACTATATGAGCCAGATTCATG<br>GTCGTCAACCAGAAAAGCAAT	G
DlREM2	0.95	1195-1245	TCTTTGATTTATATATATAAGTCAAAACT<br>ACATATAATATAGGCCTAGGC	A
	0.96	1217-1267	CAAAACTACATATAATATAGGCCTAGGC<br>TTTGTCCTTTATTTTATCTATT	T
DlREM3	0.93	1775-1825	TCTAGATGTGTTTATATGGGGAACTTAG<br>TTTGTGGAATTGCACTATTGAA	C
	0.94	356-406	GTGACTTGGTTAAATGGGCCGCAAAAAG<br>CCCAAGGAATGGGTCCAATGGT	G
	0.85	428-478	ATCAGGTCGCTATAAAAAGTTCGCTGTG<br>AAGTGCGCGTTGTTTCTTATAA	T
DlREM4	0.92	506-556	CGTTAGGTGATATATCAGCTCGCCGTGA<br>TTAATCCAGTTGACCTTGTAGA	A
	0.98	717-767	CAGCTCTCGTTATAAATATGGGTGCAAA<br>TTCATTGTAAAACAGACATAGA	C
	0.94	990-1040	ATATATATATATATATATATATTTGAAAATT<br>AACACCAACCGAATAAGAGAT	A
	0.92	398-448	GATCTCTGTTTATAAAGGGGTAAAATAT<br>AATTAAATATAATTAATCTGAT	T
DlREM5	0.91	1001-1051	TAAAATTGAATATATAAAGAGAGAAAA<br>GATATTTTTAATATTCAATTTTG	T
	0.97	1742-1792	TTTTATTGGCTAAAAAGGTGGGTTTTTTC<br>GTCTATATTTTCTTTTGGGTA	C
	0.91	1880-1930	TTTAATTGCGTAAAAAGGTGGGTTTTTTT<br>TTTTTTTTTATTTTGGAGGGG	T
	0.98	622-672	TTAGGGTTTTTAAAAAAATGGATCTAGAT<br>ATTATCTAGATCCATCCCTTAA	C
DlREM6	1	712-762	ATTTTGAGTATATATAAATTGTTACTGCA<br>GCCTCCTCTGCAGATATTCCT	A
	0.84	1692-1742	GATTTACCTGATAAAAAAATCGAGATTC<br>AATCGCTAATTTACTTTTCAAG	A
DlREM7	0.99	44-94	AGATATATATATATATATATGTATAAGA<br>GTAATGCTGAACTTCCCAAAAA	T
	0.96	52-102	ATATATATATATGTATAAGAGTAATGCT	A

			GAACTTCCCAAAAATTTTGTCC	
	0.93	651-701	CATTCATATAAATATAAATGGTTTACCTT CACATTATTTCACATTTAGAA	A
	0.83	1114-1164	TGCACGGGTCAATAAGAGCGCAAAGGG TATTTTAAGTTTTAACACCTGAT	A
	0.96	1590-1640	ATGTAGTATATATAAGAAGTTGTCTAAA ATCAAATGGATAAAAAAATACA	A
	0.88	1233-1283	AATTAAAATAAAAAATGAGAGAAGAGT CAAGAGGCTGTCCAATTTTAAAA	A
	0.95	1440-1490	AATTTAAAATAAAAATAAAGGGCCAAA GTTTATAAAAAAATAATAAAATA	T
DlREM8	0.83	1591-1641	GTAGATAGCATTATAAGGGGTGTGTAAA TATATCTAAGTCATGTTCTCAA	A
	0.94	1605-1655	AAGGGGTGTGTAAATATATCTAAGTCAT GTTCTCAAATTCAAATCCAATC	A
	0.89	1919-1969	TAAAGAAAATTAAAAAGCTCAGACAAA CCATATCACACTTTTCTCAGTCT	T
DlREM9	0.93	1796-1846	TCTAGATGTGTTTATATGGGGAACTTAG TTTGTAGAATTGCACTACTGAA	C
DlREM10	0.89	61-111	CAATGTTTTCTAAAAAAATTGGGAAATT TCGTAAAGAAGTTGGATGATGG	T
	0.94	846-896	CAAACCAATAAATAAAAGCGAAGTTTTA TGAGTTTAGGCCTGAAGGTGTA	T
DlREM11	0.95	717-767	AAATATAATATATATAATGCTAGAGGAT TCATGCCACAGTTGACTAAAAA	T
	0.85	1765-1815	TGTCTATCTGTAAAACCCCGTGAGAGAG AGAAAGGTGGCCTATTCATCAA	T
DlREM12	0.83	1427-1477	TAATATTACTTATAAATTGTGGCTCTTGG ATTGATTGTAAGGGTAGTTGT	G
	0.84	1527-1577	TCAAACCCATTAAATAACAGGGACTCTT AAAAAAAAAAAAACTAAAGAAT	A
DlREM13	0.93	308-358	GCAAGAGATGTAAAATAGAAGAGCTGG ATCTGATCGATAAAGGTGATGCT	A
	0.96	368-418	AGGGTGAGCTTATAAAAGCTAGACATAA AGTTGAGGCACATTCTTCAGAT	T
	0.88	900-950	AGTGTTGTTATATAAAAGGATTGGCTGC ACTTATAAGGGAGAAGAATTTG	G
	0.81	1932-1982	AGCCTGAGAGTATATGAAAGGATGAGG TAGCTACAACAACACCAATTTAA	A
DlREM14	0.8	32-82	TTCTCTCGTATAATTTAAAAGCTGGTATG GCATCTGATTGGTCTGAAAAG	G
	0.97	993-1043	ATGTTATGTATATATATATACATGTAAT ATTTTTTCAAATTTTATTATAT	T
	0.95	1325-1375	TATTGTCACATATATAGAGGTCATGCAT	T

			GCGTGTCAAAATTACAAGTACC	
	0.95	1527-1577	TGACGTCACATATATAGGGGCTATGCAT<br>GTGAAAATTACGACTAGTTTAG	A
	0.98	1655-1705	CTGTAGTGTATATATGTTCACCGTAGAA<br>AGTGCCTGTGTAACTAAATGAG	A
	0.93	1933-1983	ATAAAATAAATATATATACCTCTGTAAG<br>TGCAACAGAACCTTCTCAATTT	T
DlREM15	0.86	911-961	ATTAAGTATTTTAAAAGGGAAGACAATT<br>ATTCTCCTAATAAGTAAATTAT	A
	0.87	1211-1261	TTTTACTCGTTTTAACAGTGGCAGTTGCA<br>GAGTGAGAGTCAATATCGGCT	A
DlREM16	0.86	1062-1112	TCCAATACAGTATAAAAGCACCTTATTT<br>TGGATTTTTTTTTTTTAAACTC	T
DlREM17	0.87	266-316	CATCAATATAAAATATAATAAATAGGCG<br>AATAAAAGGAGCAGGTGCCATC	A
	1	276-326	AAATATAATAAATAGGCGAATAAAAGG<br>AGCAGGTGCCATCAATTCCTAAA	A
	0.94	284-334	TAAATAGGCGAATAAAAGGAGCAGGTG<br>CCATCAATTCCTAAATCTGCTAA	A
	1	1305-1355	TATATATATATATATATATGAAAGTAGG<br>GATGGTTTTTTCTATTTTGTGA	T
	0.9	1699-1749	TCCAAAAAAGGATAAAAAGACTGTGGG<br>TCCCTGAGTTGCCACCCGAGTCA	A
DlREM18	0.97	551-601	TGAAAGTGTATATATATGTTCAAGAAAT<br>GATTAGTTCGTGTTCCAATAGG	T
	0.99	614-664	TCATGGTGCATATATATCCTTGGCGAAT<br>AAAGCTAGTATGCATGTGTGCA	C
	0.84	1051-1101	TATGATTAGGCTTAAATAAACTGCAAAC<br>AGCTGTGATTTCAATCTTTCAG	A
	0.9	1820-1870	CTAACAACATATAAGATCTCCGAGTAAC<br>CACTAACCAGACAATTTAAACA	A
DlREM19	0.96	362-412	GAGATTCCCAAATAAATTGGGGGGAATG<br>AAATTCTCATTCTACAACCTTA	T
	0.94	701-751	TTTATATCGATAAATATGATGCCTTGAC<br>AGCGAAGGTGACAGAGTTTTGA	A
	0.98	1054-1104	TATATATATATATATATATATTCTTTGAA<br>TGCTGACAAGCATGAGCCTTG	A
	0.9	1121-1171	GGGCTTTGTAAATAAAATTTGGGAATTT<br>TTCACAGGGTTGTTGATGAGGG	T
	0.82	1391-1441	AGAATGGTCATAAAAAATCCACAAGAA<br>TATAATGGTTAAACCTGTGATCT	C
	0.85	1556-1606	AATCACTGTCTATATATGTTCTCTTTTCA<br>AATAATTATGACAGTTCATAA	C
	0.81	1662-1712	CTCACATATGTATAATTTTGCCAAAATC	T

			AGGCTCTCTATGTAATTTATCC	
	0.92	1791-1841	TTGAAAAAAGTATAAATAAAGCATAAA<br>GTACAGAGATGACATTCGGCCTC	A
	1	1902-1952	TATTATGTATATATATAAATGTGTTTTCA<br>TTGTTCAATCCATCTTTTACA	A
	0.83	60-110	TATGTCCGTCTTTAAAATATGGGAAGTT<br>TAACTTCCATCAGACTAGGAGT	G
	0.94	334-384	GAGTTGTCTCAATAAATGTCGAACTTAA<br>CACACAAGCATGCTTAAGTATG	C
DlREM20	0.86	449-499	TAAAGAAATAAATAAGAGCAAAAAAAT<br>GGTGATCCCTATCCTTGTCCCAA	C
	0.97	538-588	TCTATTACTATTTAAAAATAGAGCAAAT<br>TACACCAGACCCTCTCAAGGTA	T
	1	617-667	ACTTTTATAATAAAAAAGCCCCACACTG<br>TTAGTGAATGTTAGTCAACTTT	A
DlREM21	0.89	943-993	TATGCAAGAATATATAATTTGGAACTTT<br>GGTCCTTTTGACATTTAAATAA	A
	0.89	605-655	TCTCATTAGGTCTAAAAAATTCATTGAA<br>ATCCCCTCCGACAGTCCACGGT	A
	0.94	691-741	TGTGATTACCATATAAATCGGAAAATCT<br>CCGACGAAAGTCATCTTCCATC	A
DlREM22	0.98	1095-1145	ATATGTGACTTTAAAAATAGTCGCATTT<br>CATATATTAGAACAAATCAAAT	C
	0.83	1263-1313	TATTGATGTATATAAATATGATTTACATC<br>TATGTAAATGTAAAAAAAAAA	A
	0.98	1622-1672	AACTTTCCTATTAAAATGATGCGGTGAT<br>AGGTAAGCTTTCACTGGTTGTC	A
	0.82	348-398	ATTCACTTCCAATAAAACCCACGCGGCG<br>ATGAAACCCACAGGCAAATATG	G
	0.97	689-739	CTAACTCGTTATATATAAACTACCAAGG<br>CACTATTTTTACAATACAAAAC	A
DlREM23	0.88	1408-1458	TTTACATCTATAAAATCGTATGAAGATC<br>CCGCATTTGATAAATTTTATTA	A
	0.82	1516-1566	TTCTTTAAAAATATATATAATGCAAAAA<br>AATAAATAAATAAACAAGAAAA	A
	0.8	1787-1837	TTCCCATCTTATATATCAGTGACAAAATT<br>ACACAAAAGACATTGTTTTAT	A
	0.94	717-767	TGTGATTACCATATAAATCGGAAAATCT<br>CCGACGAAAGTCATCTTCCATC	A
DlREM24	0.98	1121-1171	ATATGTGACTTTAAAAATAGTCGCATTT<br>CATATATTTGAACAAATCAAAT	C
	0.86	1289-1339	TATCGATGTATATAAATATGATTTACAT<br>CTGTGTAAATGTAAAAAGAAAA	A
DlREM25	0.82	230-280	TAAAAAATATTAAAAAATTCCTCTAAAA	A

			TAGTATCAATAGAGAAAAATAT	
	0.96	326-376	AGGATTTTGTTATATGAGGGGCAAATTG<br>TACAAACCTTACAAGGTTGGAT	A
DlREM26	0.92	1849-1899	TAACCTCTTCTATATACATGGACATCACT<br>ATTTTTGTCCCTCTATTTTCA	T
	0.8	547-597	TAAACAAATGAAAAAAAGTCGAACTTC<br>GTCTTCATCCTCCACCTTAGAGG	A
DlREM27	0.94	693-743	CAATTTTGTCTATAATAGCAAACAAATT<br>CTTGGGGCCCCCGTCTCAACTG	G
	0.99	1470-1520	TGATGTTGTCTATATAAGAAACATCCCA<br>TATACCTATGGCGTCCACATAA	G
	0.95	1860-1910	GCCCCCTATCTATATATATACTTGTTTGC<br>ATGCACACATAAACCTTGTAT	A
DlREM28	0.88	1613-1663	ATGCTTTAATAAATAAAAGATCCAACAA<br>CTCAGACTCTTCAAATTTTAGC	A
	0.8	652-702	AAGGCAGAACTATAATAGAGGAAGAAA<br>AGGCAGAGGAAGAGGCCGAAATG	G
DlREM29	0.92	1478-1528	TTCTTGTACATATAAAGGTTCAAACACT<br>TGTCAATTTGTCAATAAGTTTC	A
	0.9	1580-1630	TTGTGATATAAATAAAACTTGCAACAAT<br>GTTGCCTTTGTTGGTTCCAGTG	G
	0.81	1834-1884	TAGTGTGTGCTGATATATGGGGACCAGC<br>ACCAGTTCTCTGATCTCATGGT	A
	0.98	629-679	CTACAATCAATATATACGGGGTACTAAT<br>CAAATAGAATACTTCCATTCAT	T
DlREM30	0.92	1563-1613	TTGATGGGTCTATAAGAGACATCATTGG<br>TTAGGCTTAATCTTTACTGTAT	T
	0.86	1850-1900	AGTCACCCTAAAAATGTCTATATATGCA<br>CACGGCAATTTAGAAGAGATAT	G
	0.99	1858-1908	TAAAAATGTCTATATATGCACACGGCAA<br>TTTAGAAGAGATATCCCATTGT	A
DlREM31	0.89	937-987	TTAGTCATTATATAAGTTTGGAGAGTAT<br>AATATGGATTTCATAAAATCTT	A
	0.85	1927-1977	TTTCTCATCATAAAAAATCCAGAGGTAC<br>TTGAAAACCTAGCTAGTTAGTA	C
	0.96	214-264	ATTAAAAATATATAAATTAAAGGGTGTG<br>CATATGATTTTAGAGAGATGGA	G
	0.98	715-765	ATAAAATATATATATATGTATATAGAGA<br>AAGATGGTAGTGATTCCTTTGT	A
DlREM32	0.81	1126-1176	TTGATTGGATTATATGAGCCAGGTTTATT<br>TATACGAATATATTACAATGA	A
	0.81	1575-1625	AGAACAACTTTAAAAACGAGAGATATA<br>ACTCACATCGTTGAGCTTGCCCA	A
	0.89	1683-1733	TTTGGATCTATCTATCTCCGCCCCCGGGA	A

			ACCTTGTTGAAAATTCCACAA	
DlREM33	0.89	786-836	ATTACAAGTATATATAATACTATCATAC<br>TTTATTGATCAGGATTCTGAGA	G
	0.97	1174-1224	CTTCCTTGTAAATAAATGTCCAGATAGC<br>TTTTATTCAGGTGGCACCAGTT	G
	0.99	1460-1510	CAAGGCGGACAATATATAGGTGGGTGCT<br>GAGCTAATAGGCATCCACTTCC	A
	0.92	1656-1706	CTAGATGTGGTTTAAATGGGGCTATAGG<br>TTTTCACCCATCAATATGCGTC	A
DlREM34	0.96	296-346	ATTAAAAATATATAAATTAAAGGGTGTG<br>CATATGATTTTAAAGAGATGAA	A
	0.81	1107-1157	TTGATTGGATTATATGAGCGAGGTTTAT<br>TTATACGAATAGATTACAATGA	A
	0.99	1557-1607	AAACGAGAGATATATATAACTCACATCG<br>TTGAGCTTGCCCAGTAACCGAG	A
	0.84	1698-1748	TGAAATGTAAAAAGCTAAAACGCCCCTG<br>CTAAGCCACTTTTGAGGCAAAA	T
	0.9	1838-1888	ATATATATATATATATATATATGAAATA<br>TATATATTATTAAAATACCTCC	A
	0.9	1893-1943	ATTTTTTATATTAAAAATAGGGTTAAGT<br>GATATTATTTTTCAAATTTTTT	C
DlREM35	0.96	132-182	ATTAAAAATATATAAATTAAAGGGTGTG<br>CATATGATTTTAGAGAGATGGA	G
	0.98	635-685	ATAAAATATATATATATGTATATAGAGA<br>AAGATGGTAGTGATTCCTTTGT	A
	0.81	1575-1625	AGAACAACTTTAAAAACGAGAGATATA<br>ACTCACATCGTTGAGCTTGCCCA	A
	0.89	1683-1733	TTTGGATCTATCTATCTCCGCCCCCGGGA<br>ACCTTGTTGAAAATTCCACAA	A
DlREM36	0.92	515-565	TCATTTTTGATATAAATGGTTCCTATGTA<br>GTTGCTTAATTACTCTTTGTT	A
	1	1272-1322	TAGGTAATATATATATATACACACACAT<br>ATGCACACACACACACATACAC	A
	0.86	1849-1899	TGATAGCAGTACATAATTAGCCACAAAT<br>CATTGCGATCCCACGAGTGTTA	A
DlREM37	0.93	166-216	AGAGGGCCCACTCTATAAAAGTTGTTGC<br>ACCATAAGAAACAATTTTTAAT	A
	0.97	1069-1119	TTTCGCTTTCTATCAAAACTGCGCGAATC<br>AGGAGACTGACATATTTAGCA	A
	0.97	1250-1300	AGTGATTTCATATATATGATGTGAGTGA<br>ATGAATTTGCTGACTAGATTTG	A
DlREM38	0.86	186-236	TTTAGTGCTTAAATAATGGTGGACAACT<br>CAAAGAAAATCTAGATTTGATA	A
	0.86	186-236	TTTAGTGCTTAAATAATGGTGGACAACT	A

			CAAAGAAAATCTAGATTTGATA	
	0.87	648-698	GGCCAATAAAAAAATATATATATTACGT GGGCCCATTAGCAATCAAACGA	A
	1	656-706	AAAAAATATATATATTACGTGGGCCCAT TAGCAATCAAACGACGCCCCGC	G
	0.92	745-795	CGAGTGACGGTATAAAGTCGACTCACTT TCTTCAGGCGAAAGAAGAGACC	A
	0.99	1124-1174	TATATACATATATATATATGTGGGTATTT CTGTTGCATTAGGGTTGGTGC	G
	0.98	1206-1256	GTTCCTAGGAAAAAAAAAGGGGTAAAG TTAAGGTCTTTATAAGTTGAAAA	A
	0.85	1868-1918	TATTCTTTGTTTTAAAAGAGAGAGATTTT CCTGACATGAGTTCTATCTCC	T
	0.8	241-291	AAGGGTTTAATATAAAACTAAGGGTGGC AATTTGGAACCTAGCATGACAG	A
	0.88	337-387	ACGGGTTAGATAAATAAACGGATCAACT TATCTAAATCGTTTACTAATCG	T
	0.99	482-532	GTGGTAGGTATATATAACCTTCAAACCC TAACCACACCACATAACTCCAC	A
	0.97	940-990	CCTAGTAGTTTATAAAACTTGCTTCTACC TGTAGCAAAGTAGTGTTGGTT	A
DlREM39	0.84	1217-1267	TAAACGGATCACTAAATATGTCGGCTAA ACAAGTTAATAAATGACCTGAT	A
	0.93	1242-1292	TAAACAAGTTAATAAATGACCTGATAAT AAACGGGTTAACCTTATCGTTT	C
	0.97	1362-1412	GTTCGTGTTATATAAAAAATAATCGTAT CGTATTCGTGTCATGTCAAACC	A
	0.89	1638-1688	AATAATTCTTTAAAATAATGGAGCCCAC TCAATAATTAACAAAATTACAC	A
	0.85	1839-1889	TTAAAAAAAAAAAAAAAGGTGCTTTTCC CTTGGATAAGTCAAAGGAAACT	A
	0.86	201-251	CAAATAAATAAATAAAAGGACATCGAG ACTACATATTTGACTTTTGGATA	C
	0.95	318-368	CTCTAAACAATAAAAAAATTGCCAAAAGC ATTGTGGCATGTCTCATGACCG	C
	0.88	1010-1060	AATTAAAATAAAAAATGAGAGAAGAGT CAAGAGGCTGTCCAATTTTAAAA	A
DlREM40	0.92	1216-1266	TAATTTAAAATAAAAATAAAGGGCATAG GTTATAAAAAAAATAATAAAAT	A
	0.87	1382-1432	AAGGGGTGTGTAAATGTAGCTAAGTCAT GTTCTCAAATTCAAACTCAATC	A
	0.98	1695-1745	TAAAGCAAATTAAAAAAGCTCAGACAA ACCATATCACACTTTTCTCAGTC	T
DlREM41	0.97	429-479	TTTGAGGATAAATATAAGAAGCTCAATA	A

			CAAAGAACAAAAAGTAAGAATA	
	0.81	995-1045	AGGATACCTATATCAAATGGACGAGCTC CACTTCAAAATGGACTTCCTCA	G
	0.87	1158-1208	ACCAGAAATAAATAAACCAGTGCTGGG GAAAGTGCTTGCCAGCACTATGT	A
	0.94	705-755	AAAAAAAGTCTATCAATGGCGGGAATAT GTGTGAGGAAAAAAATTATTAA	A
DlREM42	0.85	1786-1836	ATTGATGAAATTTAAAAAGCCGATATGG TGTTTGATTTGTCTGAAAAGTT	C
	0.95	1884-1934	ATTTCATGAACAAAAAAAAGGTGGTGG GATGAGCAGAATGAAGTGTTGAA	A

Supplementary Text S1: Protein sequence of AtB3s

>AtARF1

MAASNHSSGKPGGVLSDALCRELWHACAGPLVTLPREGERVYYFPEGHMEQ
LEASMHQGLEQQMPSFNLPSKILCKVINIQRRAEPETDEVYAQITLLPELDQSE
PTSPDAPVQEPEKCTVHSFCKTLTASDTSTHGGFSVLRRHADDCLPPLDMSQQ
PPWQELVATDLHNSEWHFRHIFRGQPRRHLLTTGWSVFVSSKKLVAGDAFIFL
RGENEELRVGVRRHMRQQTNIPSSVISSHSMHIGVLATAAHAITTGTIFSVFYK
PRTSRSEFIVSVNRYLEAKTQKLSVGMRFKMRFEGEEAPEKRFSGTIVGVQEN
KSSVWHDSEWRSLKVQWDEPSSVFRPERVSPWELEPLVANSTPSSQPQPPQRN
KRPRPPGLPSPATGPSGPVTPDGVWKSPADTPSSVPLFSPPAKAATFGHGGNKS
FGVSIGSAFWPTNADSAAESFASAFNNESTEKKQTNGNVCRLFGFELVENVNV
DECFSAASVSGAVAVDQPVPSNEFDSGQQSEPLNINQSDIPSGSGDPEKSSLRSP
QESQSRQIRSCTKVHMQGSAVGRAIDLTRSECYEDLFKKLEEMFDIKGELLES
TKKWQVVYTDDEDDMMMVGDDPWNEFCGMVRKIFIYTPEEVKKLSPKNKL
AVNARMQLKADAEENGNTEGRSSSMAGSR*

>AtARF2

MASSEVSMKGNRGGDNFSSSGFSDPKETRNVSVAGEGQKSNSTRSAAAERAL
DPEAALYRELWHACAGPLVTVPRQDDRVFYFPQGHIEQVEASTNQAAEQQMP
LYDLPSKLLCRVINVDLKAEADTDEVYAQITLLPEANQDENAIEKEAPLPPPPR
FQVHSFCKTLTASDTSTHGGFSVLRRHADECLPPLDMSRQPPTQELVAKDLHA
NEWRFRHIFRGQPRRHLLQSGWSVFVSSKRLVAGDAFIFLRGENGELRVGVRR
AMRQQGNVPSSVISSHSMHLGVLATAWHAISTGTMFTVYYKPRTSPSEFIVPF
DQYMESVKNNYSIGMRFKMRFEGEEAPEQRFTGTIVGIEESDPTRWPKSKWR
SLKVRWDETSSIPRPDRVSPWKVEPALAPPALSPVPMPRPKRPRSNIAPSSPDSS
MLTREGTTKANMDPLPASGLSRVLQGQEYSTLRTKHTESVECDAPENSVVWQ
SSADDDKVDVVSGSRRYGSENWMSSARHEPTYTDLLSGFGTNIDPSHGQRIPF
YDHSSSPSMPAKRILSDSEGKFDYLANQWQMIHSGLSLKLHESPKVPAATDAS
LQGRCNVKYSEYPVLNGLSTENAGGNWPIRPRALNYYEEVVNAQAQAQARE
QVTKQPFTIQEETAKSREGNCRLFGIPLTNNMNGTDSTMSQRNNLNDAAGLT
QIASPKVQDLSDQSKGSKSTNDHREQGRPFQTNNPHPKDAQTKTNSSRSCTK
VHKQGIALGRSVDLSKFQNYEELVAELDRLFEFNGELMAPKKDWLIVYTDEE

NDMMLVGDDPWQEFCCMVRKIFIYTKEEVRKMNPGTLSCRSEEEAVVGEGS
DAKDAKSASNPSLSSAGNS*
>AtARF3
MGGLIDLNVMETEEDETQTQTPSSASGSVSPTSSSSASVSVVSSNSAGGGVCL
ELWHACAGPLISLPKRGSLVLYFPQGHLEQAPDFSAAIYGLPPHVFCRILDVKL
HAETTTDEVYAQVSLLPESEDIERKVREGIIDVDGGEEDYEVLKRSNTPHMFC
KTLTASDTSTHGGFSVPRRAAEDCFPPLDYSQPRPSQELLARDLHGLEWRFRH
IYRGQPRRHLLTTGWSAFVNKKKLVSGDAVLFLRGDDGKLRLGVRRASQIEG
TAALSAQYNQNMNHNNFSEVAHAISTHSVFSISYNPKASWSNFIIPAPKFLKVV
DYPFCIGMRFKARVESEDASERRSPGIISGISDLDPIRWPGSKWRCLLVRWDDI
VANGHQQRVSPWEIEPSGSISNSGSFVTTGPKRSRIGFSSGKPDIPVSEGIRATDF
EESLRFQRVLQGQEIFPGFINTCSDGGAGARRGRFKGTEFGDSYGFHKVLQGQ
ETVPAYSITDHRQQHGLSQRNIWCGPFQNFSTRILPPSVSSSPSSVLLTNSNSPN
GRLEDHHGGSGRCRLFGFPLTDETTAVASATAVPCVEGNSMKGASAVQSNHH
HSQGRDIYAMRDMLLDIAL*
>AtARF4
MEFDLNTEIAEVEEEENDDVGVGVGGGTRIDKGRLGISPSSSSSCSSGSSSSSSS
TGSASSIYSELWHACAGPLTCLPKKGNVVVYFPQGHLEQDAMVSYSSPLEIPK
FDLNPQIVCRVVNVQLLANKDTDEVYTQVTLLPLQEFSMLNGEGKEVKELGG
EEERNGSSSVKRTPHMFCKTLTASDTSTHGGFSVPRRAAEDCFAPLDYKQQRP
SQELIAKDLHGVEWKFRHIYRGQPRRHLLTTGWSIFVSQKNLVSGDAVLFLRD
EGGELRLGIRRAARPRNGLPDSIIEKNSCSNILSLVANAVSTKSMFHVFYSPRAT
HAEFVIPYEKYITSIRSPVCIGTRFRMRFEMDDSPERRCAGVVTGVCDLDPYR
WPNSKWRCLLVRWDESFVSDHQERVSPWEIDPSVSLPHLSIQSSPRPKRPWAG
LLDTTPPGNPITKRGGFLDFEESVRPSKVLQGQENIGSASPSQGFDVMNRRILD
FAMQSHANPVLVSSRVKDRFGEFVDATGVNPACSGVMDLDRFPRVLQGQEIC
SLKSFPQFAGFSPAAAPNPFAYQANKSSYYPLALHGIRSTHVPYQNPYNAGNQ
SSGPPSRAINFGEETRKFDAQNEGGLPNNVTADLPFKIDMMGKQKGSELNMN
ASSGCKLFGFSLPVETPASKPQSSSKRICTKVHKQGSQVGRAIDLSRLNGYDD
LLMELERLFNMEGLLRDPEKGWRILYTDSENDMMVVGDDPWHDFCNVVWK
IHLYTKEEVENANDDNKSCLEQAALMMEASKSSSVSQPDSSPTITRV*
>AtARF5
MMASLSCVEDKMKTSCLVNGGGTITTTTSQSTLLEEMKLLKDQSGTRKPVIN
SELWHACAGPLVCLPQVGSLVYYFSQGHSEQVAVSTRRSATTQVPNYPNLPSQ
LMCQVHNVTLHADKDSDEIYAQMSLQPVHSERDVFPVPDFGMLRGSKHPTEF
FCKTLTASDTSTHGGFSVPRRAAEKLFPPLDYSAQPPTQELVVRDLHENTWTF
RHIYRGQPKRHLLTTGWSLFVGSKRLRAGDSVLFIRDEKSQLMVGVRRANRQ
QTALPSSVLSADSMHIGVLAAAAHATANRTPFLIFYNPRACPAEFVIPLAKYRK
AICGSQLSVGMRFGMMFETEDSGKRRYMGTIVGISDLDPLRWPGSKWRNLQ
VEWDEPGCNDKPTRVSPWDIETPESLFIFPSLTSGLKRQLHPSYFAGETEWGSL
IKRPLIRVPDSANGIMPYASFPSMASEQLMKMMMRPHNNQNVPSFMSEMQQN
IVMGNGGLLGDMKMQQPLMMNQKSEMVQPQNKLTVNPSASNTSGQEQNLS
QSMSAPAKPENSTLSGCSSGRVQHGLEQSMEQASQVTTSTVCNEEKVNQLLQ
KPGASSPVQADQCLDITHQIYQPQSDPINGFSFLETDELTSQVSSFQSLAGSYK

QPFILSSQDSSAVVLPDSTNSPLFHDVWDTQLNGLKFDQFSPLMQQDLYASQN
ICMSNSTTSNILDPPLSNTVLDDFCAIKDTDFQNHPSGCLVGNNNTSFAQDVQS
QITSASFADSQAFSRQDFPDNSGGTGTSSSNVDFDDCSLRQNSKGSSWQKIATP
RVRTYTKVQKTGSVGRSIDVTSFKDYEELKSAIECMFGLEGLLTHPQSSGWKL
VYVDYESDVLLVGDDPWEEFVGCVRCIRILSPTEVQQMSEEGMKLLNSAGIN
DLKTSVS*
>AtARF6
MRLSSAGFNPQPHEVTGEKRVLNSELWHACAGPLVSLPPVGSRVVYFPQGHS
EQVAASTNKEVDAHIPNYPSLHPQLICQLHNVTMHADVETDEVYAQMTLQPL
NAQEQKDPYLPAELGVPSRQPTNYFCKTLTASDTSTHGGFSVPRRAAEKVFPP
LDYSQQPPAQELMARDLHDNEWKFRHIFRGQPKRHLLTTGWSVFVSAKRLVA
GDSVLFIWNDKNQLLLGIRRANRPQTVMPSSVLSSDSMHLGLLAAAAHAAAT
NSRFTIFYNPRASPSEFVIPLAKYVKAVYHTRVSVGMRFRMLFETEESSVRRY
MGTITGICDLDPTRWANSHWRSVKVGWDESTAGERQPRVSLWEIEPLTTFPM
YPSPFPLRLKRPWPPGLPSFHGLKEDDMGMSMSSPLMWDRGLQSLNFQGMG
VNPWMQPRLDTSGLLGMQNDVYQAMAAAALQDMRGIDPAKAAASLLQFQ
NSPGFSMQSPSLVQPQMLQQQLSQQQQQLSQQQQQQQQLSQQQQQQLSQQQ
QQQLSQQQQQQLSQQQQQQAYLGVPETHQPQSQAQSQSNNHLSQQQQQVV
DNHNPSASSAAVVSAMSQFGSASQPNTSPLQSMTSLCHQQSFSDTNGGNNPIS
PLHTLLSNFSQDESSQLLHLTRTNSAMTSSGWPSKRPAVDSSFQHSGAGNNNT
QSVLEQLGQSHTSNVPPNAVSLPPFPGGRECSIEQEGSASDPHSHLLFGVNIDSS
SLLMPNGMSNLRSIGIEGGDSTTLPFTSSNFNNDFSGNLAMTTPSSCIDESGFL
QSSENLGSENPQSNTFVKVYKSGSFGRSLDISKFSSYHELRSELARMFGLEGQ
LEDPVRSGWQLVFVDRENDVLLLGDDPWPEFVSSVWCIKILSPQEVQQMGKR
GLELLNSAPSSNNVDKLPSNGNCDDFGNRSDPRNLGNGIASVGGSFNY*
>AtARF7
PVNKYDRDALLASDMGLKLNRQPNEFFCKTLTASDTSTHGGFSVPRRAAEKIF
PALDFSMQPPCQELVAKDIHDNTWTFRHIYRGQPKRHLLTTGWSVFVSTKRLF
AGDSVLFIRDGKAQLLLGIRRANRQQPALSSSVISSDSMHIGVLAAAAHANAN
NSPFTIFYNPRWAAPAEFVVPLAKYTKAMYAQVSLGMRFRMIFETEECGVRR
YMGTVTGISDLDPVRWKNSQWRNLQIGWDESAAGDRPSRVSVWDIEPVLTPF
YICPPPFFRPRFSGQPGMPDDETDMESALKRAMPWLDNSLEMKDPSSTIFPGL
SLVQWMNMQQQNGQLPSAAAQPGFFPSMLSPTAALHNNLGGTDDPSKLLSF
QTPHGGISSSNLQFNKQNQQAPMSQLPQPPTTLSQQQQLQQLLHSSLNHQQQ
QSQSQQQQQQQQLLQQQQQLQSQQHSNNNQSQSQQQQQLLQQQQQQQLQQ
QHQQPLQQQTQQQQLRTQPLQSHSHPQPQQLQQHKLQQLQVPQNQLYNGQQ
AAQQHQSQQASTHHLQPQLVSGSMASSVITPPSSSLNQSFQQQQQQSKQLQQ
AHHHLGASTSQSSVIETSKSSSNLMSAPPQETQFSRQVEQQQPPGLNGQNQQT
LLQQKAHQAQAQQIFQQSLLEQPHIQFQLLQRLQQQQQQQFLSPQSQLPHHQ
LQSQQLQQLPTLSQGHQFPSSCTNNGLSTLQPPQMLVSRPQEKQNPPVGGGV
KAYSGITDGGDAPSSSTSPSTNNCQISSSGFLNRSQSGPAILIPDAAIDMSGNLV
QDLYSKSDMRLKQELVGQQKSKASLTDHQLEASASGTSYGLDGGENNRQQN
FLAPTFGLDGDSRNSLLGGANVDNGFVPDTLLSRGYDSQKDLQNMLSNYGG
VTNDIGTEMSTSAVRTQSFGVPNVPAISNDLAVNDAGVLGGGLWPAQTQRMR

TYTKVQKRGSVGRSIDVNRYRGYDELRHDLARMFGIEGQLEDPQTSDWKLV
YVDHENDILLVGDDPWEEFVNCVQSIKILSSAEVQQMSLDGNFAGVPVTNQA
CSGGDSGNAWRGHYDDNSATSFNR*
>AtARF8
MKLSTSGLGQQGHEGEKCLNSELWHACAGPLVSLPSSGSRVVYFPQGHSEQV
AATTNKEVDGHIPNYPSLPPQLICQLHNVTMHADVETDEVYAQMTLQPLTPEE
QKETFVPIELGIPSKQPSNYFCKTLTASDTSTHGGFSVPRRAAEKVFPPLDYTL
QPPAQELIARDLHDVEWKFRHIFRGQPKRHLLTTGWSVFVSAKRLVAGDSVIFI
RNEKNQLFLGIRHATRPQTIVPSSVLSSDSMHIGLLAAAAHASATNSCFTVFFH
PRASQSEFVIQLSKYIKAVFHTRISVGMRFRMLFETEESSVRRYMGTITGISDLD
SVRWPNSHWRSVKVGWDESTAGERQPRVSLWEIEPLTTFPMYPSLFPLRLKRP
WHAGTSSLPDGRGDLGSGLTWLRGGGGEQQGLLPLNYPSVGLFPWMQQRLD
LSQMGTDNNQQYQAMLAAGLQNIGGGDPLRQQFVQLQEPHHQYLQQSASH
NSDLMLQQQQQQQASRHLMHAQTQIMSENLPQQNMRQEVSNQPAGQQQQL
QQPDQNAYLNAFKMQNGHLQQWQQQSEMPSPSFMKSDFTDSSNKFATTASP
ASGDGNLLNFSITGQSVLPEQLTTEGWSPKASNTFSEPLSLPQAYPGKSLALEP
GNPQNPSLFGVDPDSGLFLPSTVPRFASSSGDAEASPMSLTDSGFQNSLYSCMQ
DTTHELLHGAGQINSSNQTKNFVKVYKSGSVGRSLDISRFSSYHELREELGKM
FAIEGLLEDPLRSGWQLVFVDKENDILLLGDDPWESFVNNVWYIKILSPEDVH
QMGDHGEGSGGLFPQNPTHL*
>AtARF9
MANRGGEYLYDELWKLCAGPLVDVPQAQERVYYFPQGHMEQLEASTQQVD
LNTMKPLFVLPPKILCNVMNVSLQAEKDTDEVYAQITLIPVGTEVDEPMSPDP
SPPELQRPKVHSFSKVLTASDTSTHGGFSVLRKHATECLPPLDMTQQTPTQELV
AEDVHGYQWKFKHIFRGQPRRHLLTTGWSTFVTSKRLVAGDTFVFLRGENGE
LRVGVRRANLQQSSMPSSVISSHSMHLGVLATARHATQTKTMFIVYYKPRTSQ
FIISLNKYLEAMSNKFSVGMRFKMRFEGEDSPERRYSGTVIGVKDCSPHWKDS
KWRCLEVHWDEPASISRPNKVSPWEIEPFVNSENVPKSVMLKNKRPRQVSEV
SALDVGITASNLWSSVLTQPHEFAQSCITSQWSSPQQCHRDANEDAKKSDWL
NNSYSVSNVAKDSTLNDQMVSPVEQKKPETTANYRLFGIDLMSSSLAVPEEKT
APMRPINISKPTMDSHSDPKSEISKVSEEKKQEPAEGSPKEVQSKQSSSTRSRTK
VQMQGVPVGRAVDLNALKGYNELIDDIEKLFDIKGELRSRNQWEIVFTDDEG
DMMLVGDDPWPEFCNMVKRIFIWSKEEVKKMTPGNQLRMLLREVETTLTTT
SKTDNHSN*
>AtARF10
MEQEKSLDPQLWHACAGSMVQIPSLNSTVFYFAQGHTEHAHAPPDFHAPRVP
PLILCRVVSVKFLADAETDEVFAKITLLPLPGNDLDLENDAVLGLTPPSSDGNG
NGKEKPASFAKTLTQSDANNGGGFSVPRYCAETIFPRLDYSAEPPVQTVIAKDI
HGETWKFRHIYRGTPRRHLLTTGWSTFVNQKKLIAGDSIVFLRSESGDLCVGI
RRAKRGGLGSNAGSDNPYPGFSGFLRDDESTTTTSKLMMMKRNGNNDGNA
AATGRVRVEAVAEAVARAACGQAFEVVYYPRASTPEFCVKAADVRSAMRIRW
CSGMRFKMAFETEDSSRISWFMGTVSAVQVADPIRWPNSPWRLLQVAWDEPD
LLQNVKRVSPWLVELVSNMPTIHLSPFSPRKKIRIPQPFEFPFHGTKFPIFSPGFA
NNGGGESMCYLSNDNNNAPAGIQGARQAQQLFGSPSPSLLSDLNLSSYTGNN

KLHSPAMFLSSFNPRHHHYQARDSENSNNISCSLTMGNPAMVQDKKKSVGSV
KTHQFVLFGQPILTEQQVMNRKRFLEEEAEAEEEKGLVARGLTWNYSLQGLE
TGHCKVFMESEDVGRTLDLSVIGSYQELYRKLAEMFHIEERSDLLTHVVYRD
ANGVIKRIGDEPFSDFMKATKRLTIKMDIGGDNVRKTWITGIRTGENGIDASTK
TGPLSIFA*
>AtARF11
MSQTSLEPLIISIIKLQILQLWLKLIAVGWNLGSNDDELYTELWKACAGPLVEV
PRYGERVFYFPQGHMEQLVASTNQGVVDQEIPVFNLPPKILCRVLSVTLKAEH
ETDEVYAQITLQPEEDQSEPTSLDPPLVEPAKPTVDSFVKILTASDTSTHGGFSV
LRKHATECLPSLDMTQPTPTQELVARDLHGYEWRFKHIFRGQPRRHLLTTGWS
TFVTSKRLVAGDAFVFLRGETGDLRVGVRRLAKQQSTMPASVISSQSMRLGV
LATASHAVTTTTIFVVFYKPRISQFIISVNKYMMAMKNGFSLGMRYRMRFEGE
ESPERIFTGTIIGSGDLSSQWPASKWRSLQIQWDEPSSIQRPNKVSPWEIEPFSPS
ALTPTPTQQQSKSKRSRPISEITGSPVASSFLSSFSQSHESNPSVKLLFQDPATER
NSNKSVFSSGLQCKITEAPVTSSCRLFGFDLTSKPASATIPHDKQLISVDSNISDS
TTKCQDPNSSNSPKEQKQQTSTRSRIKVQMQGTAVGRAVDLTLLRSYDELIKE
LEKMFEIEGELSPKDKWAIVFTDDEGDRMLVGDDPWNEFCKMAKKLFIYPSD
EVKKMRSKSLLGDKGTIVNLESDQRTVHV*
>AtARF12
MESGNVVNAQPELSGIIDGSKSYVYEQLWKLCAGPLCDIPKLGEKVYYFPQG
HIELVETSTREELNELQPICDLPSKLQCRVIAIHLKVENNSDETYAEITLMPDTT
QVVIPTQNENQFRPLVNSFTKVLTASDTSAHGGFFVPKKHAIECLPSLDMSQPL
PAQELLAIDLHGNQWRFNHNYRGTPQRHLLTTGWNAFTTSKKLVAGDVIVFV
RGETGELRVGIRRARHQQGNIPSSIVSIDCMRHGVVASAKHAFDNQCMFTVV
YKPRSSKFIVSYDKFLDAVNNKFNVGSRFTMRLEGDDFSERRCFGTIIGVSDFS
PHWKCSEWRSLEVQWDEFTSFPGPKKVSPWDIEHLMPAINVPRSFLLKNKRL
REVNEIGSSSSHLLPPILTQGQENEQLSVASPMNISLRYRDATEDAMNPSKLLM
SYPVQPMPKLNYNNQMVTEMEENITTKTGTNFRLFGVTLDTPPVIKDPIEEIGS
EISKLTEGKKFGLSQTLRSPTEIQNKQFSSSRTCTKVQMQGVTIGRAVDLSVLN
GYDQLILELEKLFDIKGQLQTRNQWEIAFTDSDEDKMLVGDDPWPEFCNMVK
KIFIQKRR*
>AtARF13
MENNGEMNAQPELSVDITKTYMYEKLWNICAGPLCVLPKPGEKVYYFPQGHI
ELIENSTRDELDHIRPIFDLPSKLRCRVVAIDRKVDKNTDEVYAQISLMPDTTEV
MTHNTTMDTRRPIVYFFSKILTASDVSLSGGLIIPKQYAIECFPPLDMSQPISTQ
NLVAKDLYGQEWSFKHVFRGTPQRHMFTSGGGWSVFATTKRLIVGDIFVLLR
GENGELRFGIRRAKHQQGHIPSSVISANCMQHGVIASVVNAFKTKCMFNVVY
KPRMQFEGKDFSEKRYDGTIIGVNDMSPHWKDSEWRSLKVQWDELSPFLRP
NQVSPWDIEHLIPSSDISQSSLKKKKHWLQLNEIGATLSNLWTCQEIGQRSMNS
PISVPEFSYPNAIEDSKFLSGLLLNHSLLAIPNENYNSDQMIQPRKEDITTEATTS
CLLFGVDLTKVHMQGVAISRAVDLTAMHGYNQLIQKLEELFDLKDELRTRNQ
WEIVFTNNEGAEMLVGDDPWPEFCNMAKRIFICSKEEIKKMKLKNKFFQPES
KALTSSDVPPNVTDN*
>AtARF14

MESGNVVNTQPELSGIIDGSKSYMYEQLWKLCAGPLCDIPKLGEKVYYFPQG
HIELVEASTREELNELQPICDFPSKLQCRVIAIQLKVENNSDETYAEITLMPDTT
QVVIPTQNQNQFRPLVNSFTKVLTASDTSVHGGFSVPKKHAIECLPPLDMSQPL
PTQEILAIDLHGNQWRFRHIYRGTAQRHLLTIGWNAFTTSKKLVEGDVIVFVR
GETGELRVGIRRAGHQQGNIPSSIVSIESMRHGIIASAKHAFDNQCMFIVVYKP
RSSQFIVSYDKFLDVVNNKFNVGSRFTMRFEGDDFSERRSFGTIIGVSDFSPHW
KCSEWRSLEVQWDEFASFPRPNQVSPWDIEHLTPWSNVSRSSFLKNKRSREV
NEIGSSSSHLLPPTLTQGQEIGQQSMATPMNISLRYRDITEDAMTPSRLLMSYP
VQPMAKLNYNNVVTPIEENITTNAVASFRLFGVSLATPSVIKDPVEQIGLEISRL
TQEKKFGQSQILRSPTEIQSKQFSSTRTCTKVQMQGVTIGRAVDLSVLNGYDQ
LILELEKLFDLKGQLQARNQWEIAFTNNEEDKMLVGEDPWPEFCNMVKKIFI
YSKEEVKNLKSRKSLSS*
>AtARF15
METGNVVNAQPELSGIIDRSKSYMYEQLWKLCAGPLCDIPKLGEKVYYFPQG
NIELVEASTREELNELQPICDLPSKLQCRVIAIHLKVENNSDETYAKITLMPDTT
QVVIPTQNENQFRPLVNSFTKVLTASDISANGVFSVPKKHAIECLPPLDMSQPL
PAQELLAIDLHGNQWSFRHSYRGTPQRHLLTTGWNEFTTSKKLVKGDVIVFV
RGETGELRVGIRRARHQQGNIPSSIVSIDCMRHGVIASAKHAFDNQCMFIVVY
KPRSSQFIVSYDKFLDAVNNKFNVGSRFTMRFEGDDLSERRYFGTIIGVSNFSP
HWKCSDWRSLEVQWDEFASFLRPNKVSPWEIEHLMPALNVPRSSFLKNKRLR
EVNEFGSSSSHLLPPILTQGQEIGQLSVASPMNISLLYRETTEDAMNPSRLLMSY
PVQPMPKRNYNNQMVTQIEENITTKAGTNFRLFGVSLATPPVIKDPIEQIGSDIS
KLTEGKKFGQSQTLRSPTKIQSKQFSSTRTCTKVQMQGVTIGRAVDLSVLNGY
DQLILELEKLFDLKGQLQTRNQWKIIFTGSDEDEMLVGDDPWPEFCNMVKRI
YIQKRR*
>AtARF16
MINVMNPMKGGTEKGLDPQLWHACAGGMVRMPPMNSKVFYFPQGHAENA
YDCVDFGNLPIPPMVLCRVLAIKYMADAESDEVFAKLRLIPLKDDEYVDHEY
GDGEDSNGFESNSEKTPSFAKTLTQSDANNGGGFSVPRYCAETIFPRLDYNAE
PPVQTILAKDVHGDVWKFRHIYRGTPRRHLLTTGWSNFVNQKKLVAGDSIVF
MRAENGDLCVGIRRAKRGGIGNGPEYSAGWNPIGGSCGYSSLLREDESNSLR
RSNCSLADRKGKVTAESVIEAATLAISGRPFEVVYYPRASTSEFCVKALDARA
AMRIPWCSGMRFKMAFETEDSSRISWFMGTVSAVNVSDPIRWPNSPWRLLQV
AWDEPDLLQNVKRVNPWLVELVSNVHPIPLTSFSPPRKKMRLPQHPDYNNLIN
SIPVPSFPSNPLIRSSPLSSVLDNVPVGLQGARHNAHQYYGLSSSDLHHYYLNR
PPPPPPPSSLQLSPSLGLRNIDTKNEKGFCFLTMGTTPCNDTKSKKSHIVLFGKL
ILPEEQLSEKGSTDTANIEKTQISSGGSNQNGVAGREFSSSDEGSPCSKKVHDA
SGLETGHCKVFMESDDVGRTLDLSVLGSYEELSRKLSDMFGIKKSEMLSSVLY
RDASGAIKYAGNEPFSEFLKTARRLTILTEQGSESVVV*
>AtARF17
MSPPSATAGDINHREVDPTIWRACAGASVQIPVLHSRVYYFPQGHVEHCCPLL
STLPSSTSPVPCIITSIQLLADPVTDEVFAHLILQPMTQQQFTPTNYSRFGRFDG
DVDDNNKVTTFAKILTPSDANNGGGFSVPRFCADSVFPLLNFQIDPPVQKLYV
TDIHGAVWDFRHIYRGTPRRHLLTTGWSKFVNSKKLIAGDSVVFMRKSADEM

FIGVRRTPISSSDGGSSYYGGDEYNGYYSQSSVAKEDDGSPKKTFRRSGNGKL
TAEAVTDAINRASQGLPFEVVFYPAAGWSEFVVRAEDVESSMSMYWTPGTRV
KMAMETEDSSRITWFQGIVSSTYQETGPWRGSPWKQLQITWDEPEILQNVKR
VNPWQVEIAAHATQLHTPFPPAKRLKYPQPGGGFLSGDDGEILYPQSGLSSAA
APDPSPSMFSYSTFPAGMQGARQYDFGSFNPTGFIGGNPPQLFTNNFLSPLPDL
GKVSTEMMNFGSPPSDNLSPNSNTTNLSSGNDLVGNRGPLSKKVNSIQLFGKII
TVEEHSESGPAESGLCEEDGSKESSDNETQLSLSHAPPSVPKHSNSNAGSSSQG
IFRSLSSTIIN*
>AtARF18
MASVEGDDDFGSSSSRSYQDQLYTELWKVCAGPLVEVPRAQERVFYFPQGH
MEQLVASTNQGINSEEIPVFDLPPKILCRVLDVTLKAEHETDEVYAQITLQPEE
DQSEPTSLDPPIVGPTKQEFHSFVKILTASDTSTHGGFSVLRKHATECLPSLDMT
QATPTQELVTRDLHGFEWRFKHIFRGQPRRHLLTTGWSTFVSSKRLVAGDAFV
FLRGENGDLRVGVRRLARHQSTMPTSVISSQSMHLGVLATASHAVRTTTIFVV
FYKPRISQFIVGVNKYMEAIKHGFSLGTRFRMRFEGEESPERIFTGTIVGSGDLS
SQWPASKWRSLQVQWDEPTTVQRPDKVSPWEIEPFLATSPISTPAQQPQSKCK
RSRPIEPSVKTPAPPSFLYSLPQSQDSINASLKLFQDPSLERISGGYSSNNSFKPE
TPPPPTNCSYRLFGFDLTSNSPAPIPQDKQPMDTCGAAKCQEPITPTSMSEQKK
QQTSRSRTKVQMQGIAVGRAVDLTLLKSYDELIDELEEMFEIQGQLLARDKWI
VVFTDDEGDMMLAGDDPWNEFCKMAKKIFIYSSDEVKKMTTKLKISSSLENE
EYGNESFENRSRG*
>AtARF19
MKAPSNGFLPSSNEGEKKPINSQLWHACAGPLVSLPPVGSLVVYFPQGHSEQV
AASMQKQTDFIPNYPNLPSKLICLLHSVTLHADTETDEVYAQMTLQPVNKYD
REALLASDMGLKLNRQPTEFFCKTLTASDTSTHGGFSVPRRAAEKIFPPLDFS
MQPPAQEIVAKDLHDTTWTFRHIYRGQPKRHLLTTGWSVFVSTKRLFAGDSV
LFVRDEKSQLMLGIRRANRQTPTLSSSVISSDSMHIGILAAAAHANANSSPFTIF
FNPRASPSEFVVPLAKYNKALYAQVSLGMRFRMMFETEDCGVRRYMGTVTGI
SDLDPVRWKGSQWRNLQVGWDESTAGDRPSRVSIWEIEPVITPFYICPPPFFRP
KYPRQPGMPDDELDMENAFKRAMPWMGEDFGMKDAQSSMFPGLSLVQWM
SMQQNNPLSGSATPQLPSALSSFNLPNNFASNDPSKLLNFQSPNLSSANSQFNK
PNTVNHISQQMQAQPAMVKSQQQQQQQQQHQHQQQQLQQQQQLQMSQQ
QVQQQGIYNNGTIAVANQVSCQSPNQPTGFSQSQLQQQSMLPTGAKMTHQNI
NSMGNKGLSQMTSFAQEMQFQQQLEMHNSSQLLRNQQEQSSLHSLQQNLSQ
NPQQLQMQQQSSKPSPSQQLQLQLLQKLQQQQQQQSIPPVSSSLQPQLSALQQ
TQSHQLQQLLSSQNQQPLAHGNNSFPASTFMQPPQIQVSPQQQGQMSNKNLV
AAGRSHSGHTDGEAPSCSTSPSANNTGHDNVSPTNFLSRNQQQGQAASVSAS
DSVFERASNPVQELYTKTESRISQGMMNMKSAGEHFRFKSAVTDQIDVSTAGT
TYCPDVVGPVQQQQTFPLPSFGFDGDCQSHHPRNNLAFPGNLEAVTSDPLYSQ
KDFQNLVPNYGNTPRDIETELSSAAISSQSFGIPSIPFKPGCSNEVGGINDSGIM
NGGGLWPNQTQRMRTYTKVQKRGSVGRSIDVTRYSGYDELRHDLARMFGIE
GQLEDPLTSDWKLVYTDHENDILLVGDDPWEEFVNCVQNIKILSSVEVQQMS
LDGDLAAIPTTNQACSETDSGNAWKVHYEDTSAAASFNR*
>AtARF20

METGNVVNAQPELSGIIDGSKSYMYEQLWKLCAGPLCDIPKLGENVYYFPQG
NIELVDASTREELNELQPICDLPSKLQCRVIAIHLKVENNSDETYAEITLMPDTT
QVVIPTQSENQFRPLVNSFTKVLTASDTSAYGGFFVPKKHAIECLPPLDMSQPL
PAQELLAKDLHGNQWRFRHSYRGTPQRHSLTTGWNEFTTSKKLVKGDVIVFV
RGETGELRVGIRRARHQQGNIPSSIVSIDCMRHGVIASAKHALDNQCIFIVVYK
PRSSQFIVSYDKFLDAMNNKFIVGSRFTMRFEGDDFSERRYFGTIIGVNDFSPH
WKCSEWRSLEVQWDEFASFSRPNKVSPWEIEHLMSALNVPRSSLLKNKRLRE
VNEFGQEIGQLSVASPMNTSLRYRDTTEDAMNPSRLLMSYPVQPMPKLNYNN
QMVTQIEENITTKAVTNFRLFGVSLAIPLVIKDPIEEIGSDISKLTEGKKFGQSQT
LRSPIEIQSKQFGSTRTCTKVQMQGVTIGRAVDLSVLNGYDQLILELEKLFDLK
GQLQTRNQWKIAFTDSDGYEMLVGDDPWPEFCKMVKKILIYSKEEVKNLKSS
KSLSS*

>AtARF21

MESGNIVNAQPKLSGIIDGSKSYMYEQLWKLCAGPLCDIPKLGENVYYFPQG
NIELVQASTREELNELQPICDLPSKLQCRVIAIHLKVENNSDEIYAEITLMPDTT
QVVIPTQSENRFRPLVNSFTKVLTASDTSAYGGFSVPKKHAIECLPPLDMSQPL
PAQEILAIDLHDNQWRFRHNYRGTPQRHSLTTGWNEFITSKKLVKGDVIVFVR
GETGELRVGIRRARHQQGNIPSSIVSIDCMRHGVIASAKHAFDNQCIFIVVYKP
RSSQFIVSYDKFLDAVNNKFNVGSRFTMRFEGDDFSERRYFGTIIGVSDFSPHW
KCSEWRSLEVQWDEFASFSRPNKVSPWEIEHLVPALNVPRSSLLKNKRLREVN
EFGSSSSHLLPPILTQGQEIGQLSVASPMNISLRYRDTTEAAMNPSRLLMSYPV
QPMPKLNYNNQMVTQIEENITTKAGTNFRLFGVTLDTPPMIKDPIKQIGSDISK
LTERKKFGQSQTLRSPIEIQSKQFSSSRTCTKVQMQGVTIGRAVDLSVLNGYDQ
LILELEKLFDIKGQLQTRNQWKIAFTDSDGYEMLVGDDPWPEFCKMVKKILIY
SKEEVKNLKSSKSLSS*

>AtARF22

MESGNIVNAQPELSGIIDGSKSYMYEQLWKLCAGPLCDIPKLGEKIYYFPQGNI
ELVEASTREELNELKPICDLPSKLQCRVIAIQLKVENNSDETYAEITLMPDTTQV
VIPTQNENQFRPLVNSFTKVLTASDTSGGFFVPKKHAIECLPPLDMSQPLPTQE
LLATDLHGNQWRFNHNYRGTPQRHLLTTGWNAFTTSKKLVAGDVIVFVRGET
GELRVGIRRAGHQQGNIPSSIISIESMRHGVIASAKHAFDNQCMFIVVYKPRSS
QFIVSYDKFLDAVNNKFNVGSRFTMRFEGDDFSERRYFGTIIGVSDFSPHWKC
SEWRNLEVQWDEFASFSRPNKVSPWEIEHLMPALNVPRPSLLKNKRLREVNEI
GSSSSHLLPPILTQGQEIGQLSVASPMNISLTYRDTTEDVMNPSRLLMSYPVQP
MPKLNYNNQMVTQIEENITTKTGTNFRLFGVSLVTPSVIKDPIEEIGSEISKLTE
GKKFGQSQTLRSPTEIQSKQFSSTRTCTKVQMQGVTIERAVDLSVLNGYDQLI
LELEELFDLKGQLQTRNQWEIAFTDSDDDKMLVGDDPWPEFCNMVKKILIFK
RGGQKLEVQ*

>AtARF23

MESGNVVNVQSELSGIIDGSKSYMYEQLWKLCAGPLCDIPKLGEKVYYFPQG
HIELVEASTREELNELQPNCDLPSKLQCRVIAIHLKVENNSDETYVEITLMPDT
TQVVIPTENENQFRPIVNSFTKVLTASDTSAQGEFSVPCKHAIECLPPLDMSQPI
PAQELIAIDLHGNQWRFKHSYRVPRGDTTGWNAFTTSKKLVVGDVIVFARGE
TGELRVGIR*

>AtVAL2
MESIKVCMNALCGAASTSGEWKKGWPMRSGDLASLCDKCGCAYEQSIFCEV
FHAKESGWRECNSCDKRLHCGCIASRFMMELLENGGVTCISCAKKSGLISMN
VSHESNGKDFPSFASAEHVGSVLERTNLKHLLHFQRIDPTHSSLQMKQEESLL
PSSLDALRHKTERKELSAQPNLSISLGPTLMTSPFHDAAVDDRSKTNSIFQLAP
RSRQLLPKPANSAPIAAGMEPSGSLVSQIHVARPPPEGRGKTQLLPRYWPRITD
QELLQLSGQYPHLSNSKIIPLFEKVLSASDAGRIGRLVLPKACAEAYFPPISLPE
GLPLKIQDIKGKEWVFQFRFWPNNNSRMYVLEGVTPCIQSMQLQAGDTVTFS
RTEPEGKLVMGYRKATNSTATQMFKGSSEPNLNMFSNSLNPGCGDINWSKLE
KSEDMAKDNLFLQSSLTSARKRVRNIGTKSKRLLIDSVDVLELKITWEEAQEL
LRPPQSTKPSIFTLENQDFEEYDEPPVFGKRTLFVSRQTGEQEQWVQCDACGK
WRQLPVDILLPPKWSCSDNLLDPGRSSCSAPDELSPREQDTLVRQSKEFKRRR
LASSNEKLNQSQDASALNSLGNAGITTTGEQGEITVAATTKHPRHRAGCSCIV
CSQPPSGKGKHKPSCTCTVCEAVKRRFRTLMLRKRNKGEAGQASQQAQSQSE
CRDETEVESIPAVELAAGENIDLNSDPGASRVSMMRLLQAAAFPLEAYLKQKA
ISNTAGEQQSSDMVSTEHGSSSAAQETEKDTTNGAHDPVN*
>AtVAL1
MFEVKMGSKMCMNASCGTTSTVEWKKGWPLRSGLLADLCYRCGSAYESSL
FCEQFHKDQSGWRECYLCSKRLHCGCIASKVTIELMDYGGVGCSTCACCHQL
NLNTRGENPGVFSRLPMKTLADRQHVNGESGGRNEGDLFSQPLVMGGDKRE
EFMPHRGFGKLMSPESTTTGHRLDAAGEMHESSPLQPSLNMGLAVNPFSPSFA
TEAVEGMKHISPSQSNMVHCSASNILQKPSRPAISTPPVASKSAQARIGRPPVEG
RGRGHLLPRYWPKYTDKEVQQISGNLNLNIVPLFEKTLSASDAGRIGRLVLPK
ACAEAYFPPISQSEGIPLKIQDVRGREWTFQFRYWPNNNSRMYVLEGVTPCIQ
SMMLQAGDTVTFSRVDPGGKLIMGSRKAANAGDMQGCGLTNGTSTEDTSSS
GVTENPPSINGSSCISLIPKELNGMPENLNSETNGGRIGDDPTRVKEKKRTRTIG
AKNKRLLLHSEESMELRLTWEEAQDLLRPSPSVKPTIVVIEEQEIEEYDEPPVF
GKRTIVTTKPSGEQERWATCDDCSKWRRLPVDALLSFKWTCIDNVWDVSRCS
CSAPEESLKELENVLKVGREHKKRRTGESQAAKSQQEPCGLDALASAAVLGD
TIGEPEVATTTRHPRHRAGCSCIVCIQPPSGKGRHKPTCGCTVCSTVKRRFKTL
MMRRKKKQLERDVTAAEDKKKKDMELAESDKSKEEKEVNTARIDLNSDPYN
KEDVEAVAVEKEESRKRAIGQCSGVVAQDASDVLGVTELEGEGKNVREEPRV
SS*
>AtVAL3
MLSSSSMSSSSLSARFCFNHECFEFKLDHCRPGWRLRSGDFVDLCDRCASAYE
QGKFCDVFHQRASGWRCCESCGKRIHCGCIASASAYTLMDAGGIECLACARK
KFALGPNFSPSPSFLFQSPISEKFKDLSINWSSSTRSNQISYQPPSCLDPSVLQFD
FRNRGGNNEFSQPASKERVTACTMEKKRGMNDMIGKLMSENSKHYRVSPFPN
VNVYHPLISLKEGPCGTQLAFPVPITTPIEKTGHSRLDGSNLWHTRNSSPLSRL
HNDLNGGADSPFESKSRNVMAHLETPGKYQVVPRFWPKVSYKNQVLQNQS
KESESVVTPLFEKILSATDTGKRLVLPKKYAEAFLPQLSHTKGVPLTVQDPMG
KEWRFQFRFWPSSKGRIYVLEGVTPFIQTLQLQAGDTVIFSRLDPERKLILGFR
KASITQSSDQADPADMHSPFEVKKSAYITKETPGVECSSGKKKSSMMITRSKR
QKVEKGDDNLLKLTWEEAQGFLLPPPNLTPSRVVIEDYEFEEYEEAPIIGKPTD

VAGFRSTCTEVEGLLISPTTTKHPRHRDGCTCIICIQSPSGIGPKHDRCCSCAVC
DTNKRRRRSLLLRREKKQMEKEDNARKLLEQLNSDNGLHQSANNSENHERH
ASPLKVQLDLNFKPEKDEESLPGSNKTTKSETLPHDDTVKSSFTSPSSSSAHSQ
NNKEDEGKLKTTTEIADTTTTSSM*
>AtFUS3
MMVDENVETKASTLVASVDHGFGSGSGHDHHGLSASVPLLGVNWKKRRMP
RQRRSSSSFNLLSFPPPMPPISHVPTPLPARKIDPRKLRFLFQKELKNSDVSSLR
RMILPKKAAEAHLPALECKEGIPIRMEDLDGFHVWTFKYRYWPNNNSRMYV
LENTGDFVNAHGLQLGDFIMVYQDLYSNNYVIQARKASEEEEVDVINLEEDD
VYTNLTRIENTVVNDLLLQDFNHHNNNNNNNSNSNSNKCSYYYPVIDDVTTN
TESFVYDTTALTSNDTPLDFLGGHTTTTNNYYSKFGTFDGLGSVENISLDDFY*
>AtLEC2
MDNFLPFPSSNANSVQELSMDPNNNRSHFTTVPTYDHHQAQPHHFLPPFSYP
VEQMAAVMNPQPVYLSECYPQIPVTQTGSEFGSLVGNPCLWQERGGFLDPRM
TKMARINRKNAMMRSRNNSSPNSSPSELVDSKRQLMMLNLKNNVQISDKKD
SYQQSTFDNKKLRVLCEKELKNSDVGSLGRIVLPKRDAEANLPKLSDKEGIVV
QMRDVFSMQSWSFKYKFWSNNKSRMYVLENTGEFVKQNGAEIGDFLTIYED
ESKNLYFAMNGNSGKQNEGRENESRERNHYEEAMLDYIPRDEEEASIAMLIG
NLNDHYPIPNDLMDLTTDLQHHQATSSSMPPEDHAYVGSSDDQVSFNDFEW
W*
>AtABI3
MKSLHVAANAGDLAEDCGILGGDADDTVLMDGIDEVGREIWLDDHGGDNN
HVHGHQDDDLIVHHDPSIFYGDLPTLPDFPCMSSSSSSSTSPAPVNAIVSSASSS
SAASSSTSSAASWAILRSDGEDPTPNQNQYASGNCDDSSGALQSTASMEIPLDS
SQGFGCGEGGGDCIDMMETFGYMDLLDSNEFFDTSAIFSQDDDTQNPNLMD
QTLERQEDQVVVPMMENNSGGDMQMMNSSLEQDDDLAAVFLEWLKNNKE
TVSAEDLRKVKIKKATIESAARRLGGGKEAMKQLLKLILEWVQTNHLQRRRT
TTTTTNLSYQQSFQQDPFQNPNPNNNNLIPPSDQTCFSPSTWVPPPPQQQAFVS
DPGFGYMPAPNYPPQPEFLPLLESPPSWPPPPQSGPMPHQQFPMPPTSQYNQF
GDPTGFNGYNMNPYQYPYVPAGQMRDQRLLRLCSSATKEARKKRMARQRR
FLSHHHRHNNNNNNNNNNQQNQTQIGETCAAVAPQLNPVATTATGGTWMYW
PNVPAVPPQLPPVMETQLPTMDRAGSASAMPRQQVVPDRRQGWKPEKNLRF
LLQKVLKQSDVGNLGRIVLPKKEAETHLPELEARDGISLAMEDIGTSRVWNM
RYRFWPNNKSRMYLLENTGDFVKTNGLQEGDFIVIYSDVKCGKYLIRGVKVR
QPSGQKPEAPPSSAATKRQNKSQRNINNNSPSANVVVASPTSQTVK*
>AtTEM1
MEYSCVDDSSTTSESLSISTTPKPTTTTEKKLSSPPATSMRLYRMGSGGSSVVL
DSENGVETESRKLPSSKYKGVVPQPNGRWGAQIYEKHQRVWLGTFNEEEEA
ASSYDIAVRRFRGRDAVTNFKSQVDGNDAESAFLDAHSKAEIVDMLRKHTYA
DEFEQSRRKFVNGDGKRSGLETATYGNDAVLRAREVLFEKTVTPSDVGKLNR
LVIPKQHAEKHFPLPAMTTAMGMNPSPTKGVLINLEDRTGKVWRFRYSYWNS
SQSYVLTKGWSRFVKEKNLRAGDVVCFERSTGPDRQLYIHWKVRSSPVQTVV
RLFGVNIFNVSNEKPNDVAVECVGKKRSREDDLFSLGCSKKQAIINIL*
>AtTEM2

MDSSCIDEISSSTSESFSATTAKKLSPPPAAALRLYRMGSGGSSVVLDPENGLET
ESRKLPSSKYKGVVPQPNGRWGAQIYEKHQRVWLGTFNEQEEAARSYDIAAC
RFRGRDAVVNFKNVLEDGDLAFLEAHSKAEIVDMLRKHTYADELEQNNKRQ
LFLSVDANGKRNGSSTTQNDKVLKTREVLFEKAVTPSDVGKLNRLVIPKQHA
EKHFPLPSPSPAVTKGVLINFEDVNGKVWRFRYSYWNSSQSYVLTKGWSRFV
KEKNLRAGDVVTFERSTGLERQLYIDWKVRSGPRENPVQVVVRLFGVDIFNV
TTVKPNDVVAVCGGKRSRDVDDMFALRCSKKQAIINAL*
>AtRAV1
MESSSVDESTTSTGSICETPAITPAKKSSVGNLYRMGSGSSVVLDSENGVEAES
RKLPSSKYKGVVPQPNGRWGAQIYEKHQRVWLGTFNEEDEAARAYDVAVHR
FRRRDAVTNFKDVKMDEDEVDFLNSHSKSEIVDMLRKHTYNEELEQSKRRRN
GNGNMTRTLLTSGLSNDGVSTTGFRSAEALFEKAVTPSDVGKLNRLVIPKHHA
EKHFPLPSSNVSVKGVLLNFEDVNGKVWRFRYSYWNSSQSYVLTKGWSRFV
KEKNLRAGDVVSFSRSNGQDQQLYIGWKSRSGSDLDAGRVLRLFGVNISPESS
RNDVVGNKRVNDTEMLSLVCSKKQRIFHAS*
>AtRAV2
MDAMSSVDESSTTTDSIPARKSSSPASLLYRMGSGTSVVLDSENGVEVEVEAE
SRKLPSSRFKGVVPQPNGRWGAQIYEKHQRVWLGTFNEEDEAARAYDVAAH
RFRGRDAVTNFKDTTFEEEVEFLNAHSKSEIVDMLRKHTYKEELDQRKRNRD
GNGKETTAFALASMVVMTGFKTAELLFEKTVTPSDVGKLNRLVIPKHQAEKH
FPLPLGNNNVSVKGMLLNFEDVNGKVWRFRYSYWNSSQSYVLTKGWSRFVK
EKRLCAGDLISFKRSNDQDQKFFIGWKSKSGLDLETGRVMR
LFGVDISLNAVVVVKETTEVLMSSLRCKKQRVL*
>AtNGA1
MMTDLSLTRDEDEEEAKPLAEEEGAREVADREHMFDKVVTPSDVGKLNRLVI
PKQHAERFFPLDSSSNEKGLLLNFEDLTGKSWRFRYSYWNSSQSYVMTKGWS
RFVKDKKLDAGDIVSFQRCVGDSGRDSRLFIDWRRRPKVPDHPHFAAGAMFP
RFYSFPSTNYSLYNHQQQRHHHSGGGYNYHQIPREFGYGYFVRSVDQRNNPA
AAVADPLVIESVPVMMHGRANQELVGTAGKRLRLFGVDMECGESGMTNSTE
EESSSSGGSLPRGGGGGGASSSSFFQLRLGSSSEDDHFTKKG
KSSLSFDLDQ*
>AtNGA2
MNQEDKEKPIEEASSSMEREHMFDKVVTPSDVGKLNRLVIPKQHAERYFPLD
NSTTNDSNKGLLLNFEDRSGNSWRFRYSYWNSSQSYVMTKGWSRFVKDKKL
DAGDIVSFQRDSCNKDKLYIDWRRRPKIPDHHHQQFAGAMFPRFYTFPHPQM
PTNYETHNLYHRFHQRDLGIGYYVRSMERSHPTAVIESVPVMMQRRAQVASM
ASRGEKRLRLFGVDMECGGGGGSVNSTEEESSSSGGSIPRGRVSMVGAGSLL
QLRLVSSDDESLVAMEAASLEDHHFFTKKGKPSLSFDLDR*
>AtNGA4
MNISTMNLDQELAEIRASSSDHTNYFYSSERREHMFDKVLTPSDVGKLNRLVI
PKQHAENFFPLEDNQNGTVLDFQDKNGKMWRFRYSYWNSSQSYVMTKGWS
RFVKEKKLFAGDTVSFYRGYIPDDNAQPERRRKIMFIDWRPRAEINFVHNINN
HNFVFGSPTYPTARFYPVTPEYSMPYRSFPPFYQNQFQEREYLGYGYGRVVN
GNGVRYYAGSPLDQHHQWNLGRSEPLVYDSVPVFPAGRVPPSAPPQPSTTKK

LRLFGVDVEESSSSGDTRGEMGVAGYSSSSPVVIRDDDQSFWRSPRGEMASSS
SAMQLSDDEEYKRKGKSLEL*
>AtNGA3
MDLSLAPTTTTSSDQEQDRDQELTSNIGASSSSGPSGNNNNLPMMMIPPPEKE
HMFDKVVTPSDVGKLNRLVIPKQHAERYFPLDSSNNQNGTLLNFQDRNGKM
WRFRYSYWNSSQSYVMTKGWSRFVKEKKLDAGDIVSFQRGIGDESERSKLYI
DWRHRPDMSLVQAHQFGNFGFNFNFPTTSQYSNRFHPLPEYNSVPIHRGLNIG
NHQRSYYNTQRQEFVGYGYGNLAGRCYYTGSPLDHRNIVGSEPLVIDSVPVV
PGRLTPVMLPPLPPPPSTAGKRLRLFGVNMECGNDYNQQEESWLVPRGEIGAS
SSSSSALRLNLSTDHDDDNDDGDDGDDDQFAKKGKSSLSLNFNP*
>AtREM1
MVESELSYEQIRLNRVEENKKRMGELNLNKLAQSLRVSSSSSSSSKPSPAKPRT
MRIPVDFSEVRRSSRAKGPPPSYKEFGLEPLERRPRRSSRRRDLLNRVYASDDA
RMYAFDRAEKLQSSLDSEYASFTKPMLQSHVTGGFWLGLPLPFCKAHMPKRD
VIMTLVDEEEEEESQAKYLAQKNGLSGGWRGFAIDHQLVDGDAVVFHLIARTT
FKVYIIRVNDDANNDSDGNEVNDDDSDGNEEDRDNDNESNEKQKETVSEGR
QLRSSGKRKRRGNNVSNLFTS*
>>AtREM17
MASSMASNDFQLPPRFFTVFVSHFSSEFMVIPVSYYDHIPHRFPKTVILRGPGG
CSWKVATEIKDDEVLFSQGWPKFVRDNTLNDGDFLTFAYNGAHIFEVSIFRGY
DACKEISEVTELEEEEEDSVISLSSEDTDTGAKSEMKNTVPEGRDKGKSKVEV
VEDSDDDEEEDSVYSESSEETETDTDSEFKVAKPTIPKSQKKGKKKEQVVESS
DDEEDEEEDSDSDYIETFGQLDIEENSISEEDSSYAPDKEDTATASFVKPKVANK
VANLKRKEAAKKRKKVDPMIKNPERYLDDPKNIHFETNVKNRLYELLVHAQL
VKDYCLRFGDYVNYIDRFGKLSAKTAKWKDQRVCIKRWMRICKRNKLKKED
RILCELLRKGTFVYAIKLHVIRGKDL*
>AtREM30
MVLNSSDLGPSRCDIRDLPAPSSTNDQGKTELARKKKVKRSNTEIEADASSSD
NSCFVALVTASNLRKDALYLPQDLTSSVGLERKYREIVVTDERERRSWALDLR
FNKSSDTFYISRGWRSFCDENGKKPGGVFVFKLVGNRETPVLSFCSTESINDGT
QGHKNNKYNCMELKSKKKRMRCRDSTSPSQNRFMTLTLTHDNLIKSRRQLPL
PFMRENGLVKPGIINLLGKDGTKWEVNLRREANGTMYLGKGWKDFTIANGL
KTGESFTLEAVLENGTRMLSLVSTQSTSDRNQQGECLKDSEKESNSTEPSSGK
RKAMINRGVRRKSFPASQNRCVTLKLTHDNLIKSRRVSSSINPCFRGYVSFISC
THIRYVIRSFMLQYLPLSFTRDNGLDKPGMIFLLGKTGRKWEANLLREASGRI
VLTGKGWKEFAMANGLKSGELFTLEAILEKGTPMLSLVNTQSTNYRSQQGEC
SRDSEKESSICAEPSRGNKKWKATNNRKERRDSSSAIQNRYVTLTLTPEDVRA
CTLILPSQFMKANGINKLGKKTLLGQNRKKWFAYLLSKSGFVALGSGWKGFC
EANGVKTGESFNLEYIDEQD
TTPVFKFCSNSVEYVKFTSLP*
>AtREM15
MVLENGFPPYPDFLKIFNSHEDSQLLVIPRSYNRYYPNPLPQTAVLKNPEGRFW
NVQWTKSQEVIISLQEGWVKFVKDNGLIDRDFLLFTYDGSRSFWVRIHRNGL
PLEPTAPIKIQEISDDEDETNGDGDPHMEEEGDTDENMIVSLSLGSSDEVGDDD

DDDYDTAICDEVNKASGSSKKGRVTRKHRDDSLASITPQIFLADPNNPFFISTS
SCSRRILVIARQVIKDYGLNFDGTVNLIDGFGELTRKVGKWKDRVVIYKWNE
MFTRNKVKQGDVIICEIIREEDVVRSIKVHFVKN*
>AtREM19
MQMDSAQNQFNKRARLFEDPELKDAKVIYPSNPESTEPVNKGYGGSTAIQSFF
KESKAEETPKVLKKRGRKKKNPNPEEVNSSTPGGDDSENRSKFYESASARKR
TVTAEERERAVNAAKTFEPTNPYFRVVLRPSYLYRGCIMYLPSGFAEKYLSGIS
GFIKLQLGEKQWPVRCLYKAGRAKFSQGWYEFTLENNIGEGDVCVFELLRTR
DFVLEVTAFRVNEYV*
>AtREM38
MKNPTLFAPKSPHFFQPILPGFKSHIKIPVKFFSKHIEGKHEGNTVYLRSYPSRR
TWKVKMEGHKLTEGWKEFVEAHDLRVGDFVVFKHKGDMLFHVTAIGPSCCE
VQYAPSRSHDRNEESDEIGESSRNEKIIEENVKTEPDQFSPDLTCFSQSVTASNL
TRDLVGIPRDFAKRYGLNIGRHEIVLMDEEGNTWESEVKSYKSGRVFIAGGWT
SLCTANKLEVGDSCTFKLLRNPKIPVFRLCSRPKAGAEARPHKRSRVQSSSGG
SRQKHDVRQKLDEEGGPSRCTRSSNKATGDQEKLQQTQSCFIFDHVAKVKQS
VEDTLNSIRRFRAKLETKEHNLEALLQETKTKL*
>AtREM39
MPRPFFHKLIFSSTIQEKRLRVPDKFVSKFKDELSVAVALTVPDGHVWRVGLRK
ADNKIWFQDGWQEFVDRYSIRIGYLLIFRYEGNSAFSVYIFNLSHSEINYHSTG
LMDSAHNHFKRARLFEDLEDEDAEVIFPSSVYPSPLPESTVPANKGYASSAIQT
LFTGPVKAEEPTPTPKIPKKRGRKKKNADPEEINSSAPRDDDPENRSKFYESAS
ARKRTVTAEERERAINAAKTFEPTNPFFRVVLRPSYLYRGCIMYLPSGFAEKYL
SGISGFIKVQLAEKQWPVRCLYKAGRAKFSQGWYEFTLENNLGEGDVCVFEL
LRTRDFVLKVTAFRVNDLLEDTGENGSIEGKCIYKIVGGNCGSRHTEVKQ*
>AtREM16
MEENCEDCMKWEEELYWTHFQTLHFTQLLLPGFHNRLVIPRKFSTHCKRKLP
QIVTLKSPSGVTYNVGVEEDDEKTMAFRFGWDKFVKDHSLEENDLLVFKFHG
VSEFEVLVFDGQTLCEKPTSYFVRKCGHAEKTKGIIDFNATSSRSPKRHFNPDD
VETTPNQQLVISPPVDNELEDLIDIDLDFDIDKILNPLLVASHTGYEQEEHINSDI
DTASAQLPVISPTSTVRVSEGKYPLSGFKKMRRELSNDNLDQKADVEMISAGS
NKKALSLAKRAISPDGFLVFMKRSHVVSKCFLTIPYKWCVKNMLITRQEVVM
QVDQTKWEMKFNIFGARGSGGISTGWKKFVQDNNLREGDVCVFEPANSETK
PLHLNVYIFRGEETERTNNVDPVYTISE*
>AtREM25
MASNPDFLWCKEERKQESFFKVLKRSDMSSEDTRAIPYDFVINFSDNELSGDM
KFRVQWGNSWKVKISKNPRFYFMEKSGWEKFVIDNALGDHEFLTFTHKGQM
SFTVKIFNKDGKEMMQPPQSRASFASSSRVKTEQDVKREEEVLVSSDSRSRGP
TTAAETNRGGSYKRKLNFGKKKAEETQTYKRTERTQNSKRTERVVSKERVYA
GEPSSSVAGFKIFISKSYIKSLAIPKPFGNYMPKEKTRVKIHHPDGEKTWKVVF
VVKERGQIFSGGWKRLCKEYPVVFGDTCKFTLITPLE
LLLVVSKP*
>AtREM24
MATNAGYLRCKEERKNESFFKVVQSINVSSENKRALPHDFSRSFTDKELSRK

MKIRAQWGNSWEVGISKNPRFYFMEKSGWEKFVRDNALGNSELLTFTHKGK
MHFTVNIFKLDGKEMMQPPQSRSFFASSSRIKTEQEENDIKEEVVVSSNRGQT
TAAESKGRKLNLGKRAAKESQSSKRTEKVVRARSDYAGASSSTAAAFTILFKQ
GYLVFLRIPNSVSKDQVPDEKTVFKIHHPNGKKSWNVVYLERFGAFSGGWRR
VVKEYPLAVGDTCKFTFIKPKELLLVVSKP*
>AtREM23
MARNSDNDMCKEERKRESFFKVLQRVDISSENMRALPYDFVRSFSNNELSRK
MKIKARWGSSWEVEICKNPRFYFMEKSGWEKFVSDNALGASEFLTFTHKGN
MRFTVNIFMQDGKEMLQPPQSMSFMASSRPPKREQGIPSLATTIAAESNGGGN
YKRKLNFEKKKAEESHNSKRTDKVFSVQRESAGASSSSVAEFSMFIKKSYLIY
MWFPKSVQSIHMPKQRTIFKIHHPNMKKSWNVVYVVSGTKSSFSAGWKGLA
QEYPLAVGDTCKFSFIKQHELILFVSKP*
>AtREM22
MGKSSNIVFDRIKEERQMLSFFKIFQRADLSSDCMRALPYSFISKVSEKDFSYK
MVIRAQWGKTWDVEVSKNPTYYYIETRGWDLFVSDNALGQNEFITFTHRGN
MVFHVNIYEQNGLEMRKPRKFQTMGPSSGIKKEEGENSLIDVKKEEESDESPG
RAEFLVRKKKTEDSKSSKKKMTRNKVKKKSKSKSKQVLDGVPEFKITIRKSY
LKFLAIPKHFVDDHIPNKSKIFTIRHPNGGSWKVLCLVREIRTIFSGGYSKLARE
FPLMVGDKCTFKLIKPFEFVLLTSKKNREKMDQCM
ID*
>AtREM18
MWATKSRRCAVKKQCCFLSLVVHRARTEKNRIQSLEIFFLKIKENRLDQISLPL
VHTSSMASGDVLPRFFTVFLSHCSSESMVIPRSYYNLLPRPLPKTAILIGTGGRF
WKVAMTSKQEQVYFEQGWGNFVADNQLKEGEFLTFVFDGHKSYEVSIYGRG
DCKETRAVIQVEEISDDTEDDNVSLHSPSNVSLDSLSNDSHHSTSNVSLRSLSN
DSLHGDAEIESDSEYSPENLPSASISVESVEVVNPTTSRQRSYKRKTIENPHLYL
DDPNNVCFETCLKLRKFELLVHAQLVKDYGLIFSDNVDYIDGYGKLTAKTTK
WADQRVCINKWQKICERNQFTENDSILCEILRNEDKVVYAIKIHIFRDAAAST*
